# Effect of diabetes medications on the risk of developing dementia, mild cognitive impairment, or cognitive decline: A systematic review and meta-analysis

**DOI:** 10.1177/13872877251319054

**Published:** 2025-02-27

**Authors:** Esther K Hui, Naaheed Mukadam, Gianna Kohl, Gill Livingston

**Affiliations:** 1 Division of Psychiatry, University College London, London, UK; 2 Division of Psychology and Language Sciences, University College London, London, UK

**Keywords:** Alzheimer's disease, cognitive decline, cognitive impairment, dementia, diabetes, prevention, risk factors

## Abstract

**Background:** Diabetes is a risk factor for dementia, but we do not know whether specific diabetes medications ameliorate this risk. **Objective:** To systematically review and meta-analyze such medication's effect on the risk of developing dementia, mild cognitive impairment (MCI), or cognitive decline. **Methods:** We searched three databases until 21 November 2023. We included randomized controlled trials (RCT), cohort, and case-control studies assessing association between antidiabetic medication and future dementia, MCI, or cognitive decline. We meta-analyzed studies separately for individual drug classes and their comparators (no medication, placebo, or another drug). We appraised study quality using the Newcastle-Ottawa Scale and Physiotherapy Evidence Database Scale. **Results:** 42 studies fulfilled inclusion criteria. Glucagon-like peptide-1 receptor agonists (GLP-1 RA) versus placebo reduced dementia risk by 53% in three RCTs (n = 15,820, RR = 0.47[0.25, 0.86]) and 27% in three case-control studies (n = 312,856, RR = 0.73[0.54, 0.99], I^2 ^= 96%). Repaglinide was superior to glibenclamide by 0.8 points on the Mini-Mental State Examination scale in another RCT. Meta-analysis of seven longitudinal studies showed glitazones (n = 1,081,519, RR = 0.78[0.76, 0.81], I^2 ^= 0%) were associated with reduced dementia risk. Metformin (n = 999,349, RR = 0.94[0.79, 1.13], I^2 ^= 98.4%), sulfonylureas (RR = 0.98[0.78, 1.22], I^2 ^= 83.3%), dipeptidyl peptidase-IV inhibitors (DPP-1V) (n = 192,802, RR = 0.86[0.65, 1.15], I^2 ^= 92.9%) and insulin (n = 571,274, RR = 1.09[0.95, 1.25], I^2 ^= 94.8%) were not. Most studies were observational and limited by confounding by indication. **Conclusions:** In people with diabetes, RCTs consistently showed GLP-RAs reduce future dementia risk. Glitazones consistently showed protective effects, without heterogeneity, suggesting potential generalizability of these results. Metformin, sulfonylureas, insulin, and DPP-1V studies had inconsistent findings. If information is available future studies should consider dosage, severity, and duration.

## Introduction

Dementia and diabetes mellitus are both public health priorities. Dementia, a global epidemic, currently affects 50 million people worldwide, with projections indicating an increase to 152 million by 2050.^1^ Currently, diabetes affects approximately 463 million individuals worldwide, with an anticipation of increase to 700 million by 2045.^2^ The numbers affected by both conditions are disproportionately higher in lower-middle-income countries, and those from minority ethnic backgrounds are at greater risk.^3–6^

Diabetes can be divided into two main categories: type 1 and type 2. Type 1 is characterized by hyperglycemia due to insulin deficiency. In most, it is an autoimmune disorder where pancreatic beta cells are destroyed, which usually develops in childhood or early adulthood and is treated with insulin therapy.^7^ On the other hand, type 2 characterized by insulin resistance in peripheral tissues and progressive dysfunction of beta pancreatic cells occurs later in life and its prevalence increases with age. A pivotal trial compared metformin as monotherapy with chlorpropamide, glyburide, and insulin in a subgroup of overweight participants led to the recommendation of metformin as a first-line glucose-lowering drug.^8^ Currently, the UK National Institute for Health and Care Excellence and American Diabetes Association recommends metformin as the first-line treatment for type 2 diabetes.^9,10^ Other medications including glucagon-like peptide-1 receptor agonists (GLP-1 RA) and sodium-glucose co-transporter 2 (SGLT2), with and without metformin based glycemic control, are also used as initial therapy for those with high risk for atherosclerotic cardiovascular disease, heart filature and/or chronic kidney disease.^9^ Diabetes is also managed through healthy eating, regular exercise, weight loss, insulin therapy and blood sugar monitoring.^11^

People with diabetes are more likely to develop dementia later in life.^12^ Poorer glycemic control and younger age diabetic onset is associated with high risk of subsequent dementia.^13,14^ Previous systematic reviews have considered the overall effect of any diabetes treatments on the risk of dementia and cognitive impairment.^15–17^ However, findings were inconsistent, and all combined treatments in their meta-analyses, which makes it difficult to evaluate the impact of an individual drug on dementia risk.^15–17^

### Objective

This review aimed to systematically review, synthesize, and meta-analyze the effect of individual antidiabetic medication for people with diabetes on the risk of subsequent dementia, mild cognitive impairment, or cognitive decline.

## Methods

We performed the systematic review using a registered, prespecified protocol in accordance with the Preferred Reporting Items for Systematic Reviews and Meta-Analysis (PRISMA). registered with the International Prospective Register of Systematic Reviews (PROSPERO) on 20 April 2023 (registration number: CRD42023414040).

### Search strategies

We identified studies using MEDLINE, Embase and Cochrane Central Register of Controlled Trials (CENTRAL) with a combination of search terms: diabetic OR diabetes OR “type 1” OR “type I” or “type II” or “type 2” from inception. We used keywords and database-specific headings in the title and abstract to conduct the search, and the MeSH terms varied among databases. Searches were restricted to peer-reviewed published articles on humans with no limits on language or date of publication. We developed the search terms in conjunction with a university research subject librarian. GL and NM reviewed the keywords, and EH performed the first search on 21 April 2023, and again on 21 November 2023.

### Inclusion criteria

- Study design: randomized controlled trials (RCTs), case-control and cohort studies;
- Participants: people with diabetes mellitus; aged ≥18 years;
- Interventions: pharmacological and non-pharmacological interventions (e.g., diet, exercise, any orally administered or injected drug at any dose) or a combination of both;
- Comparisons: treatment as usual, placebo or another active treatment;
- Outcomes: Primary outcome—incidence of mild cognitive impairment or dementia was the diagnosed by validated criteria or by clinicians.^18–20^ Secondary outcome—decline in cognitive scores. Change in cognitive scores measured with cognitive tests.

### Exclusion criteria

- People with dementia at the beginning of the study.

### Selection process and data extraction

EH screened the titles and abstracts and obtained the relevant full-text articles. EH and GK assessed the full text articles against the inclusion criteria. GL and NM resolved any disagreements between the two reviewers. We recorded the reasons for excluding studies. If the study did not include all relevant data, we contacted the authors directly to request further information.

EH and GK extracted the following information from the included papers, where possible:

- Participant characteristics: sex, age, education (level and years of education), baseline cognitive function, cognitive diagnostic status, duration of cognitive symptoms, ethnicity, socioeconomic status;
- Intervention characteristics: description of the intervention, description of the control condition, frequency of the dose of treatment, duration of the treatment;
- Methodological characteristics: trial design, number of participants, outcome measure used, duration of follow-up from randomization, duration of the following measured from the end of treatment.

For dichotomous outcomes, we extracted the number of participants with each outcome at the end of treatment for each trial. For continuous outcomes, we extracted the number of participants and the mean and standard deviation of the change from baseline to post-treatment. If a study included more than one measure of cognitive function, we chose the most frequently used measure in the other included studies for comparison. We contacted authors of included studies for any relevant data that was not in the paper.

### Quality assessment

Two reviewers, EH and GK, independently appraised the included papers. For trials, we used the Physiotherapy Evidence Database (PEDro) scale.^21^ We used the Newcastle-Ottawa Criteria to assess case-control and cohort studies.^22^ To resolve discrepancies between the two reviewers, GL, NM and EH discussed and identified reasons so ratings was then consistent.

The PEDro is an 11-item scale designed for evaluation of RCTs and controlled clinical trials, incorporating internal validity (blinding, attrition, study design, allocation concealment, baseline differences) and statistical information sufficiency intention-to-treat analysis (ITT) (between-group and point measures). We scored each criterion as “1” for yes, “0” for no or “unclear” where there was insufficient detail in the study after we contacted the authors. The scale had a maximum score of 10, where the higher the score, the better the quality; five was the cut-off point for high quality according to the manual. The Newcastle-Ottawa Criteria scale assesses non-randomized studies in three areas: selection, comparability, and outcomes.^22^ With a maximum score of nine, previous studies considered high quality as a total score of ≥6.^23^

### Meta-analysis

We meta-analyzed studies together which adjusted for sex and age and divided them into two groups: (1) studies that used the same diabetic medications with no drug or (2) diabetes medication with any other drug. We converted odds ratios into risk ratios.^24–26^ We did not include meta-analyze papers that grouped multiple drug classes together, i.e., oral hypoglycemic agents. We converted the confidence intervals using the same formula (see Supplemental Text for details).^24–26^ We assumed HR and RR would be similar for this study as the prevalence of dementia outcome was low (<10%), meaning that the value of RR and HR would be similar.^27^ Using a random effects model on R Studio (version 4.3.2), we pooled results from two or more studies with the same comparator to conduct a meta-analysis. We also described the findings narratively. Within meta-analyses, we noted the heterogeneity of methods, outcomes and if there were at least 10 papers included in the meta-analysis, we conducted a meta-regression was conducted. For the sensitivity analysis, we stratified studies based on study design, where possible, and analyzed studies with Alzheimer's disease as an outcome. We assessed heterogeneity between studies using I^2^ statistics, which calculates the percentage of variability due to treatment effect heterogeneity beyond chance in random effect meta-analysis, e.g., I^2^ between 75% to 100% suggests considerable heterogeneity.^28^

## Results

### Study selection

**Figure 1.**
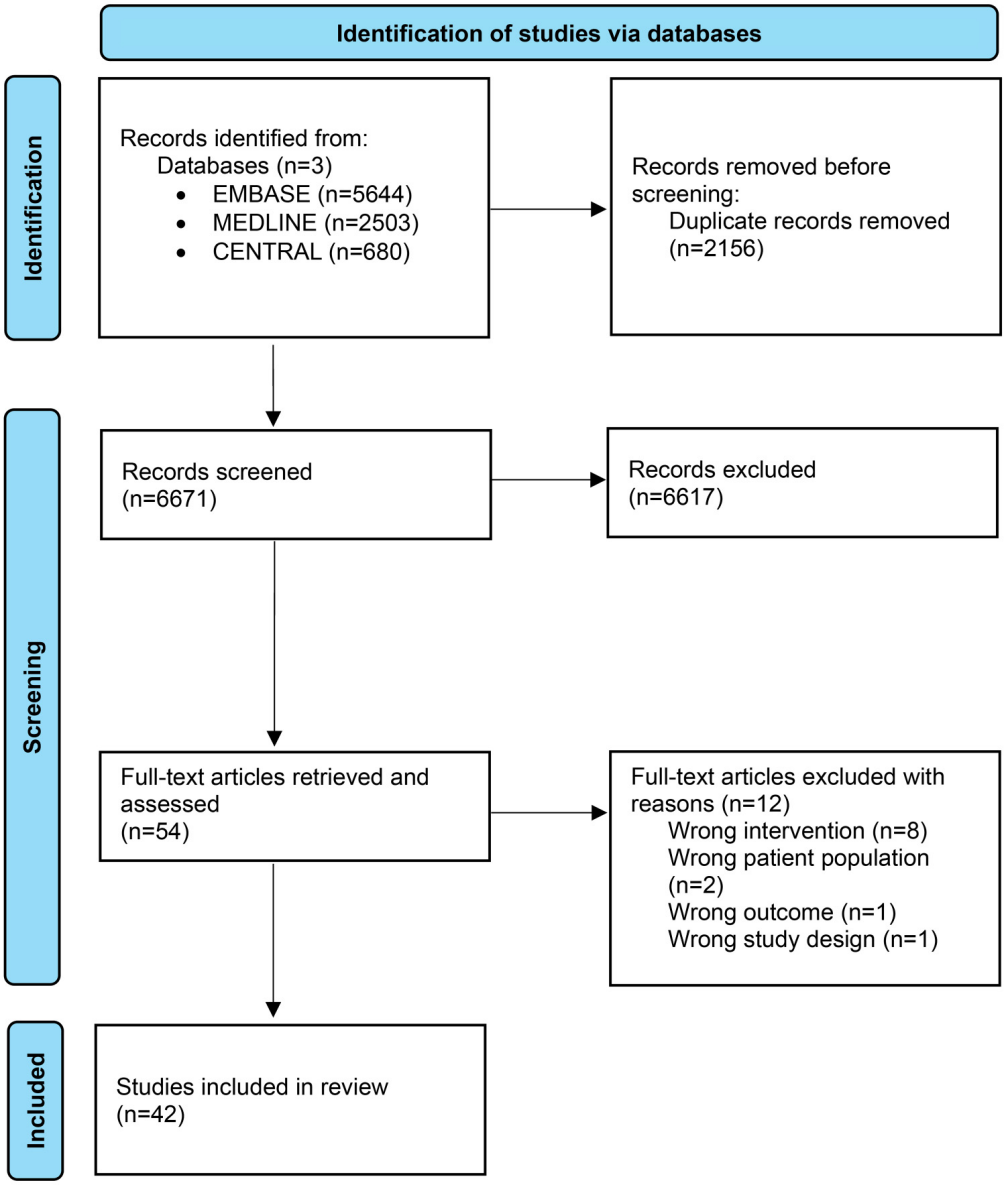
PRISMA diagram of study identification and selection.

We found 8221 studies from the search, of which 36 fulfilled inclusion criteria (see PRISMA diagram, Figure 1). An additional six studies were added from an updated search conducted on 21 November 2023, taking the total number of included studies to 42.

### Study characteristics

Ten were from Europe,^29–38^ 14 North America,^39–52^ 17 Asia,^53–68^ and two in Oceania.^69,70^ None were from low- and middle-income countries (LMIC). While all studies reported information on sex, only seven reported on minority ethnic backgrounds.^41–44^ Five of the seven divided participants based on white, black, or other populations, where the majority were white.^40–44^ Of the three studies that reported on socioeconomic status,^38,42,57^ two included similar number of people from low to high socioeconomic quintiles,^38,42^ and the third had more black people from lower SES than the white population.

All studies reported pharmacological interventions. These comprised 31 metformin,^30–32,34–36,38,41–45,47,51–56,58,59,61–64,66–71^ 12 glitazones,^30–32,36,45,47,55,59,62,63,65,66^ 10 sulfonylureas,^30,32,34,36,45,54,55,66,72,73^ nine insulin,^30,32,36,39,40,47,60,66^ three GLP-1 RA.^30,33,36^ Two included oral hypoglycemic agents,^40,60^ which could include multiple classes of drugs, such as biguanide, sulfonylureas, and glitazones. There was one study of each of meglitinide, glucosidase, combination of sulfonylureas and repaglinide, gibenclamide.^29,36,47,55^ We conducted 10 meta-analyses which included 17 studies.^30,32,33,35,36,40,45,53–55,58,60,62–66^

### Study quality

The overall quality of the included studies was high. The mean scores of the included cohort studies with dementia and cognitive decline as outcomes were seven and five, respectively. Average scores for case-control studies were seven, and RCTs were nine. All cohort studies had less than 80% follow-up, and the high drop-out rates were likely to introduce bias. Only four cohort studies had follow-up durations long enough (≥10) for outcomes to occur likely to introduce bias, and only four cohort studies had follow-up long enough (≥10 years) for outcomes to occur,^39,55,58^ and six included predominately male participants (see Tables 1–3; Supplemental Tables 1–3).^40–43,45,60^

**Table 1.** Study characteristics of included randomized controlled trials (all-cause dementia).

Study	Country	Sample size	Female (%)	Mean age (SD)	Mean/median/range follow-up years	Intervention	Comparator	Diagnostic criteria	Treatment effect (RR[95%CI]/meanΔ)	Study quality
Norgaard (2022)^33^	Denmark	15,820	35.64%	64.7 (7.3)	1. LEADER (liraglutide vs. placebo): median = 3.8 years.	GLP-1 RAs	Placebo (secondline diabetes medication)	Standardized Medical Dictionary for Regulatory Activities (MedDRA, version 21.1) for identifying dementia related adverse events using narrow scope search terms for dementia	GLP-1 RA: RR = 0.47 (0.25, 0.86)	8,10,7
					2. SUSTAIN-6 (semaglutide vs. placebo): median = 2.1 years.					
					3. PIONEER (semaglutide (oral) vs. placebo): median = 1.3 years.					

CI: confidence intervals, DPP-4i: dipeptidyl peptidase-4 inhibitors, GLP-1 RA: glucagon-like peptide-1 receptor agonists, LEADER: Liraglutide Effect and Action in Diabetes Evaluation of Cardiovascular Outcome Results trial, RR: relative risks, PIONEER: peptide innovation for early diabetes treatment, SD: standard deviation, SUSTAIN-6: semaglutide in subjects with type 2 diabetes.

**Table 2.** Study characteristics of included cohort studies (all-cause dementia).

Study	Country	Sample size	Female (%)	Intervention	Comparator	Mean age (SD)	Mean/median/range follow-up years	Diagnostic criteria	Treatment effect (RR[95%CI])	Study quality
Alkabbani (2023)^39^	Canada	33,092	39.2%	Insulin	Non-insulin use	57.1 (7.7)	6.1 years (mean)	Validated algorithm that requires one hospitalization code, 3 physician claim codes (at least 30 days apart in a 2-year period), or a prescription filled for cholinesterase inhibitor	1. Insulin: RR = 1.39 (1.05, 1.86)	8
Chen (2023)^68^	Taiwan	62,768	46.7%	Metformin	Non-use of metformin	Missing	5 years (mean)	ICD-9-CM. CDR, Cognitive Abilities Screening Instrument, MMSE	1.Metformin: RR = 0.72 (0.60, 0.85)	6
Chou (2017)^65^	Taiwan	19,203	50.5%	Pioglitazone	Non-use of pioglitazone	Missing	5 years (range)	ICD-9-CM 250.xx	1. Pioglitazone: RR = 0.77 (0.62, 0.96)	7
Heneka (2015)^31^	Germany	145,928	59.5%	Pioglitazone, rosiglitazone, and metformin	Non-use of corresponding drug	Missing	4.3 years (mean)	ICD-10 codes G30, G31, G 31.82, G23.1, F00, F01, F02, F03, F05.1	1. Pioglitazone < 8 calendar quarters: RR = 1.16 (0.87, 1.55)	6
									2. Pigolitazone ≥ 8 calendar quarters: RR = 0.53 (0.30, 0.94)	
									3. Rosiglitazone: RR = 0.84 (0.60, 1.19)	
									4. Metformin: RR = 0.97 (0.91, 1.03)	
									5. Insulin: RR = 1.61 (1.46, 1.77)	
Hsu (2011)^54^	Taiwan	25,393	51.6%	Metformin and SU (monotherapy and combination therapy)	No antidiabetics medication	Missing	7 years (range)	ICD-9-CM: A210, A222, or ICD 9-CM codes: 290.0, 290.1, 294.1, 331.0–331.2, or 331.7–331.9	1. SU: RR = 0.86 (0.73, 1.03)	7
									2. Metformin: RR = 0.76 (0.58, 0.98)	
									3. SU and metformin: RR = 0.65 (0.57, 0.75)	
Huang (2023)^67^	Taiwan	736,473	51.4%	Metformin	Non-use of corresponding drug	62.0 (8.8)	3 and 5 years (range)	3 or more outpatient visits for a dementia diagnosis within 1 year, according to the ICD-9-CM codes 290, 294.1, 331.0, and 331.82 and the ICD-10-CM codes F00-F03, F05.1, G30.0, G30.1, G30.8, and G30.9	1. Metformin: RR = 0.94 (0.92, 0.97)	8
Kim (2020)^56^	Korea	73,718	49.2%	Metformin	Non-use of metformin	Missing	12.4 years (mean)	ICD-10 codes: F00-F003, G30, G31.0, G31.1, G31.9, G31.82	1. Metformin (men): low, RR = 0.90 (0.68, 1.19); moderate, 0.75 (0.57, 0.98); high, 0.44 (0.32, 0.61)	7
									2. Metformin (women): RR = 0.79 (0.65, 0.97); moderate, 0.60 (0.49, 0.75); high, 0.45 (0.36, 0.57)	
Kim (2019)^72^	Korea	15,104	56.3%	SU, DPP-4i	Comparing to SU/DPP-4i use	75.4 (5.0)	10 years (range)	ICD-10 codes: F00, F01, F02, F03, F04, F05, G30, or G31	1. DPP-4i vs. SU: RR = 0.54 (0.40, 0.73)	5
Kim (2019)^55^	Korea	278,290	59.7%	SU, meglitinide, glucosidase, biguanide, ^a^thiazolidinedione, DPP-4i; both monotherapy and combination therapy	No antidiabetics medication	73.4 (6.5)	13 years (range)	ICD-10 codes: AD: F00; vascular dementia: F01; dementia in other diseases classified elsewhere: F02; unspecified dementia: F03; AD: G30	1. SU: monotherapy, RR = 1.0 8 (1.02, 1.15); combination therapy, RR = 0.77 (0.70, 0.80)	8
									2. Meglitinide: monotherapy, RR = 0.93 (0.70, 1.25); combination therapy, RR = 0.87 (0.81, 0.93)	
									3. Glucosidase: monotherapy, RR = 1.02 (0.86, 1.21); combination therapy, RR = 0.83 (0.79, 0.86)	
									4. Metformin: monotherapy, RR = 0.67 (0.60, 0.72); combination therapy, RR = 0.75 (0.73, 0.77)	
									5. Thiazolidinedione: monotherapy, RR = 0.76 (0.55, 1.04); combination therapy, RR = 0.82 (0.77, 0.87)	
									6. DPP-4i: monotherapy, RR = 0.31 (0.12, 0.82); combination therapy, RR = 0.48 (0.45, 0.51)	
Kuan (2017)^58^ ^b^	Taiwan	9302	49.5%	Metformin	Non-use of metformin	64.7 (9.7)	12 years (range)	ICD-9-CM codes: 290.0–290.4, 294.1, 294.2, 331.0–331.1	1. Metformin (dementia): RR = 1.66 (1.35, 2.04)	8
Ma (2014)^60^	China	634	46.8%	Insulin, oral hypoglycemic agents	No antidiabetics medication	75.3 (5.9)	4 years (range)	National Institute of Neurological and Communicative Disorders and Stroke-Alzheimer's Disease and Related Disorders Association WorkGroup, National Institute of Neurological Disorders and Stroke-Association Internationale pour la Rechercheet l’Enseignement en Neurosciences, DSM-IIIR criteria. Two examining physicians independently made a preliminary diagnosis and a third opinion was obtained in cases of disagreement	1. Insulin: RR = 1.013 (0.972, 1.056)	4
									2. Oral hypoglycemic agents: RR = 0.929 (0.890, 0.961)	
Orkaby (2017)^51^ ^b^	US	17,200	1.0%	Metformin	Sulfonylureas	74.0 (5.9)	5 years (mean)	ICD-9 codes: 290.x, 291.2, 294.1, 294.11, 331.x (except 331.83 [MCI]), 333.0, 333.4, 797, 332.0, 294.8, 046.1, and 046.3	1. Metformin: RR = 0.93 (0.87, 1.00)	7
Parikh (2011)^40^	US	377,838	2.1%	Insulin, oral hypoglycemic agents	Non-use of corresponding drug (unclear if other drugs are used)	75.5 (6.1)	2 years (mean)	ICD-9-CM codes	1. Insulin: RR = 1.02 (0.983, 1.067)	6
									2. Oral hypoglycemic agents: RR = 0.940 (0.909, 0.972)	
Salas (2019)^41^ ^b^	US	127,178	8.6%	Metformin	Unclear	62.7 (9.1)	9 years (range)	ICD-9-CM codes: 290.0–290.4, 294.1, 294.2, 331.0–331.1, 331.2, 331.82	1. VHA: RR = 1.04 (0.95, 1.13)	4
									2. KPW: RR = 0.81 (0.51, 1.28)	
Samaras (2020)^70^	Australia	123	55.3%	Metformin	Non-use of metformin	78.8 (4.8)	6 years (range)	DSM-IV/V criteria. Multidisciplinary consensus panel with old age psychiatrist, neuropsychiatrists, neuropsychologists	1. Metformin: RR = 4.14 (1.16, 9.60)	6
Scherrer (2019)^42^	US	73,761	3.2%	Metformin	Use of SU	60.9 (8.5)	5 years (range)	ICD-9-CM codes ≥2 in any 12-month period inpatient, outpatient, or Medicare data	1. White: metformin vs. SU, RR = 0.96 (0.9, 1.03)	6
									2. African American: metformin vs. SU, RR = 0.73 (0.60, 0.89)	
Scherrer (2019)^43^ ^b^	US	86,053	90.9%	Metformin, SU	Use of SU	61.1 (8.7)	6.4 years (mean)	ICD-9-CM codes	1. VHA: Metformin vs. SU, RR = 0.93 (0.87, 0.99)	6
									2. KPW: Metformin vs. SU, RR = 0.89 (0.74, 1.07)	
Shi (2019)^44^	US	5528	2%	Metformin	Non-use of metformin	63.2 (10.9)	6 years (range)	ICD-9-CM codes: 290.0–290.43, 294.8, 294.1	1.Dementia, ≤1 year: RR = 0.88 (0.64, 1.21)	4
									2. 1–2 year: RR = 1.02 (0.72, 1.44)	
									3. 2–4 years: RR = 0.55 (0.38, 0.79)	
									4. > 4 years: RR = 0.22 (0.13, 0.37)	
						
Tang (2022)^45^	US	559,106	3.1%	Metformin, SU, TZD	Use of metformin as monotherapy	65.7 (8.7)	6.8 years (mean)	ICD-9 and ICD-10	1. SU: RR = 1.12 (1.09, 1.15)	6
									2. TZD: RR = 0.78 (0.75, 0.81)	
									3. MET and SU: RR = 1.14 (1.11, 1.18)	
									4. MET and TZD: RR = 0.89 (0.86, 0.93)	
									5. SU and TZD: RR = 1.04 (1.01, 1.08)	
Tseng (2018)^63^	Taiwan	22,022	43.2%	Pioglitazone, metformin	Non-use of pioglitazone	58.7 (9.1)	2 years (range)	ICD-9-CM: A210 or A222, or as ICD-9-CM codes of 290.0, 290.1, 290.2, 290.4, 294.1, 331.0–331.2, or 331.7–331.9	1. Pioglitazone: RR = 0.716 (0.545, 0.940)	7
									2. Metformin/pioglitazone: RR = 0.802 (0.580, 1.109)	
Tseng (2019)^64^	Taiwan	31,352	57.9%	Metformin	Non-use of metformin	63.5 (10.2)	10 years (range)	ICD-9-CM: A210 or A222, or as ICD-9-CM codes of 290.0, 290.1, 290.2, 290.4, 294.1, 331.0–331.2, or 331.7–331.9.	1. Metformin: RR = 0.707 (0.632, 0.791)	8
Tseng (2019)^62^	Taiwan	10,096	45.5%	Rosiglitazone, metformin	Non-use of rosiglitazone (and metformin)	61.2 (10.0)	4.8 years (mean)	ICD-9-CM: A210 or A222, or as ICD-9-CM codes of 290.0, 290.1, 290.2, 290.4, 294.1, 331.0–331.2, or 331.7–331.9.	1. Rosiglitazone: RR = 0.895 (0.696, 1.151)	7
									2. Metformin ever-use/Rosiglitazone: RR = 0.931 (0.677, 1.279)	
									3. Metformin never-use/Rosiglitazone: RR = 0.823 (0.535, 1.267)	
Wu (2023)^48^	Canada	106,903	44.2%	SGLT2 inhibitors, DPP-4i	SGLT2 inhibitors/DPP-4i	73.6 (6.2)	2.8 years (mean)	Validated algorithm that requires hospitalization with dementia record from Discharge Abstract Database, or 3 physician claims (earliest claim) for dementia in the OHIP database at least 30 days apart in a 2-year period, or cholinesterase inhibitor prescription	1. SGLT2 inhibitors vs. DPP-4 inhibitors: RR = 0.80 (0.71, 0.89)	7
Wu (2023)^49^	Canada	34,700	48.9%	Metformin	No diabetes medication	72.5 (4.8)	6.8 years (mean)	Validated algorithm that requires 3 OHIP physician claims (first claim) for dementia at least 30 days apart in a two-year period, 1 hospitalization with a dementia record, or dispensing of a cholinesterase inhibitor	1. Metformin: RR = 1.05 (0.96, 1.15)	8
Wu (2023)^73^	Canada	144,836	46.0%	SU	DPP-4i	73.5 (5.2)	4.8 years (mean)	Validated algorithm that requires 3 OHIP physician claims (first claim) for dementia at least 30 days apart in a two-year period, 1 hospitalization with a dementia record, or dispensing of a cholinesterase inhibitor	1. Metformin: HR = 1.09 (1.04, 1.15)	7
Xu (2022)^37^	UK	495,942	54.5%	Glucosamine	Non-use of Glucoasmine	56.5 (8.19)	11 years (median)	ICD-10 codes F00-F03, and G30-G31	1. Glucosamine: RR = 0.84 (0.79, 0.89)	6
Zheng (2023)^38^	UK	210,237	45.5%	Metformin	No diabetes medication	67.0(10.5)	5 years (median)	Medcodes in CPRD; had a dementia diagnosis based on International ICD codes in linked database; at least one dementia-specific drug prescription	1. Metformin: RR = 0.90 (0.84, 0.96)	7
Zimmerman (2023)^52^	US	12,220	46.5%	Metformin	Non-use of metformin	60.6 (9.0)	5 years (range)	ICD-9 and ICD-10	1.Metformin: RR = 1.21 (1.12, 1.30)	7

CDR: Clinical Dementia Rating scale, CI: confidence intervals, CM: clinical modifications, CPRD: Clinical Practice Research Datalink, DPP-4i: dipeptidyl peptidase-4 inhibitors, DSM: diagnostic and statistical manual, ICD: International Classification of Diseases, MMSE: Mini-Mental State Examination, OHIP: Ontario Hospital Insurance Plan, RR: relative risks, SD: standard deviation, SGLT2: sodium/glucose cotransporter 2, SU: sulfonylureas, TZD: Thiazolidinediones, VHA: veteran health affairs, KPW: Kaiser Permanente Washington.

a Metformin is the only biguanide in Korea.

b Emulated trial.

**Table 3.** Study characteristics of included case-control studies (all-cause dementia).

Study	Country	Sample size	Study design	Female (%)	Mean age (SD)	Mean/range follow-up years	Intervention	Comparator	Diagnostic criteria	Treatment effect (RR[95%CI])	Study quality
Bohlken (2018)^30^	Germany	16,552	56.2%	79.9 (6.9)	79.9 (6.9)	5 years (range)	Metformin, SU, DPP-4i, GLP-1 RA, SGLT2 inhibitors, TZD, insulin	Non-use of corresponding drug	ICD-10 codes: F01, F03, G30	1. Metformin: RR = 0.96 (0.88, 1.04)	7
										2. TZD: RR = 0.81 (0.69, 0.95)	
										2. SU: RR = 1.03 (0.96, 1.10)	
										3. DPP-4i: RR = 0.99 (0.95, 1.07)	
										4. Glucagon-like-peptide 1: RR = 0.81 (0.69, 0.95)	
										5. SGLT2 inhibitors: RR = 0.92 (0.73, 1.15)	
										6. Insulin: RR = 1.31 (1.22, 1.40)	
Wium-Andersen (2019)^36^	Denmark	176,250	54.2%	Missing	Missing	17 years (range)	Insulin, metformin, SU and glinides combined, TZD, DPP-4i, GLP-1 RA, SGLT2 inhibitors and acarbose	Non-use of corresponding drug	ICD-10 codes: F00-F04 and G30	1. Insulin: RR = 1.00 (0.94, 1.54)	8
										2. Metformin: RR = 0.88 (0.84, 0.93)	
										3. SU: RR = 1.03 (0.98, 1.08)	
										4. DPP-4i: RR = 0.74 (0.69, 0.91)	
										5. GLP-1 inhibitors: RR = 0.54 (0.48, 0.62)	
										6. SGLT2 inhibitors: RR = 0.47 (0.34, 0.63)	
										7. Acarbose: RR = 1.01 (0.85, 1.19)	
Lu (2018)^59^ ^a^	Taiwan	51,415	51.6	72.5 (5.9)	72.5 (5.9)	14 years (range)	Metformin, pioglitazone	SU, acarbose, meglitinide, insulin, metformin	A210, A222; or ICD-9-CM codes: 290.0, 290.1, 294.1, 331.0–331.2, 331.7–331.9	1. Metformin and pioglitazone (SU as reference): RR = 0.57 (0.36, 0.93)	5
										2. Metformin and pioglitazone (acarbose as reference): RR = 0.68 (0.40, 1.11)	
										3. Metformin and pioglitazone (meg as reference): RR = 0.70 (0.43, 1.15)	
										4. Metformin and pioglitazone (insulin as reference): RR = 0.98 (0.44, 2.08)	
Norgaard (2022)^33^	Denmark	120,054	52.6%	52.6%	Missing	Missing	GLP-1 agonists	Second line treatments (i.e., insulin, SU, DPP-4i, meglitinides)	Diagnosis of dementia in the National Patient Register or first-ever prescription for approved dementia specific treatment in the National Prescription Register (cholinesterase inhibitors and memantine)	1.GLP1-RA: RR = 0.89 (0.86, 0.93)	7

CI: confidence intervals, CM: clinical modifications, DPP-4i: dipeptidyl peptidase-4 inhibitors, GLP-1 RA: glucagon-like peptide-1 receptor agonists, ICD: International Classification of Diseases, RR: relative risks, SD: standard deviation, SGLT2: sodium/glucose cotransporter 2, SU: sulfonylureas, TZD: Thiazolidinediones.

a Nested case-control study and emulated trial.

Table 1 and Supplemental Table 1 describe three RCTs, where Norgaard et al. (2021) pooled findings from three individual RCTs of Glucagon-like peptide-1 receptor agonists which all had dementia as an outcome. Table 2 and Supplemental Table 2 includes 30 cohorts, where five controlled for diabetes severity or duration.^47,48,52,61,74^ Three out of the eight case-control studies described in Table 3 and Supplemental Table 3 had Alzheimer's disease instead of dementia as an outcome.^32,35,53^

### Glucagon-like peptide-1 receptor agonists (GLP-1 RA)

Of the four studies (N = 338,557) that included GLP-1 RA as a treatment, two were case-control studies, one was an RCT, and one included both a case-control study and pooled three double-blind RCTs.^30,33,36,50^ Norgaard et al. pooled findings from three RCTs, and found that GLP-RA significantly reduced the rate of dementia compared with second line diabetes treatments (RR = 0.47[0.25, 0.86]).^33^ For these trials, the first-line medication, which was metformin, was not included as a comparator. Cukierman-Yaffe et al.^50^ evaluated risk of cognitive decline as an outcome in a RCT. GLP-1 RA users was associated with significantly less cognitive decline than treatment as usual, where participants could take up to two non-GLP-1 RA diabetes medications (RR = 0.86[0.79, 0.95]).^50^

**Figure 2.**
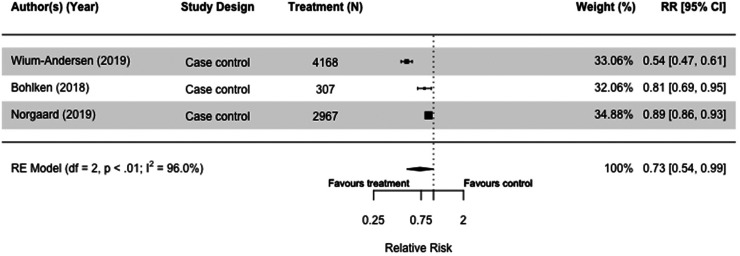
Forest plot of the effect of glucagon-like peptide 1 receptor agonists (GLP-1 RA) versus with those not taking GLP-1 RA (but on other or no diabetes medication(s)) on all-cause dementia in case-control studies.

We meta-analyzed the three case-control studies of GLP-1 RA users versus those on other second-line diabetes medication and found a reduced dementia risk (RR = 0.73[0.54, 0.99], I^2 ^= 96%) (Figure 2).^30,33,36^

### Glitazones

**Figure 3.**
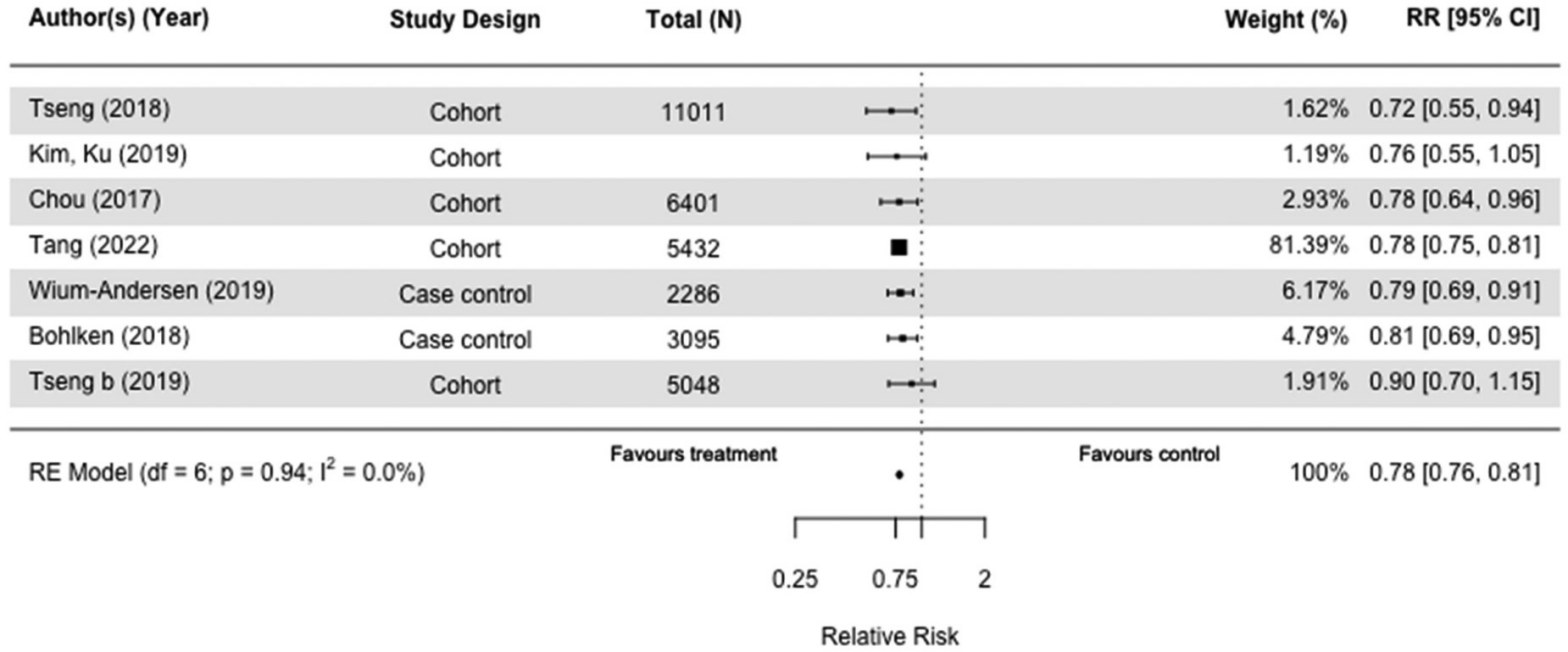
Forest plot of the effect of glitazones on versus those not taking glitazones (but on other or no diabetes medication(s)) on all-cause dementia in case-control and cohort studies.

We meta-analyzed nine of the 13 studies involving glitazones also known as thiazolidinediones (N = 1,404,830), of which three were case-control studies,^30,32,36^ and six were cohort studies.^45,55,62,63,65,66^ The use of glitazones was significantly associated with reduction of dementia risk with low heterogeneity when compared to those taking no or any other diabetes medication(s) (RR = 0.78[0.76, 0.81] I^2 ^= 0%) in both case-control and cohort studies (Figure 3).^30,36,45,55,62,63,65^ This significant reduction in dementia risk was also observed when we stratified by study design (Supplemental Figures 3 and 4).

When we included AD as an outcome in the sensitivity analysis, the results were almost identical (RR = 0.78[0.75, 0.81], I^2 ^= 0%) (Supplemental Figure 2).^30,32,36,45,55,62,63,65,66^

### Sulfonylureas

**Figure 4.**
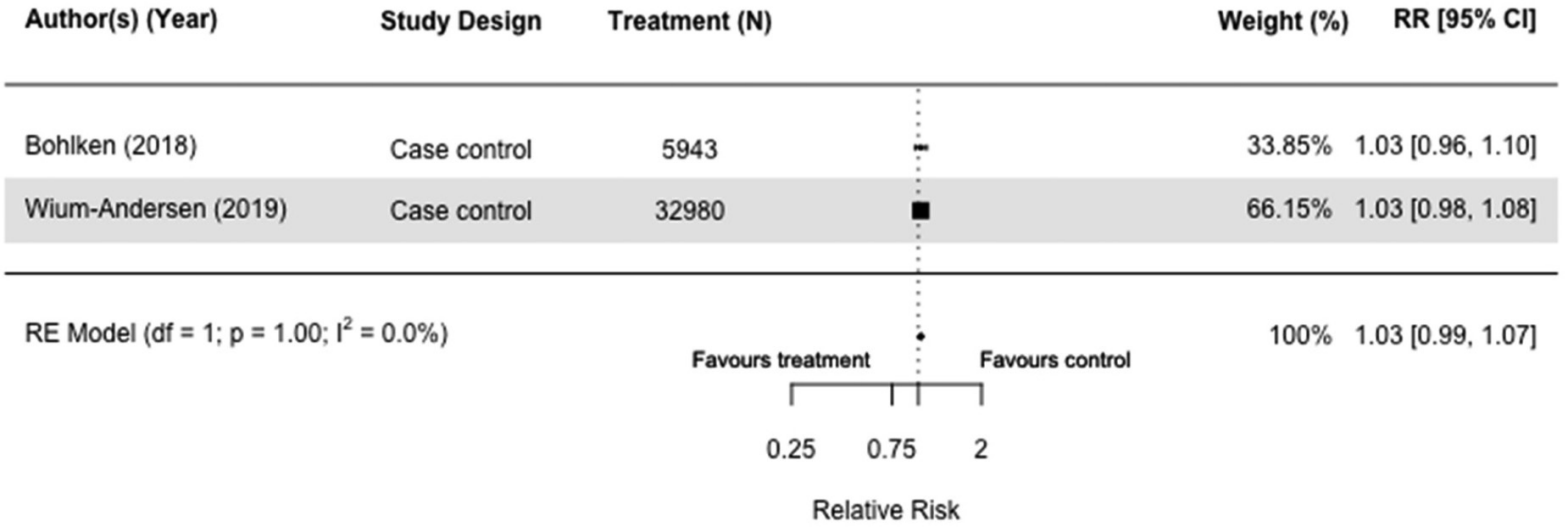
Forest plot of the effect of sulfonylureas (SU) versus those not taking SU (but on other diabetes medication(s)) on all-cause dementia in case-control studies.

**Figure 5.**
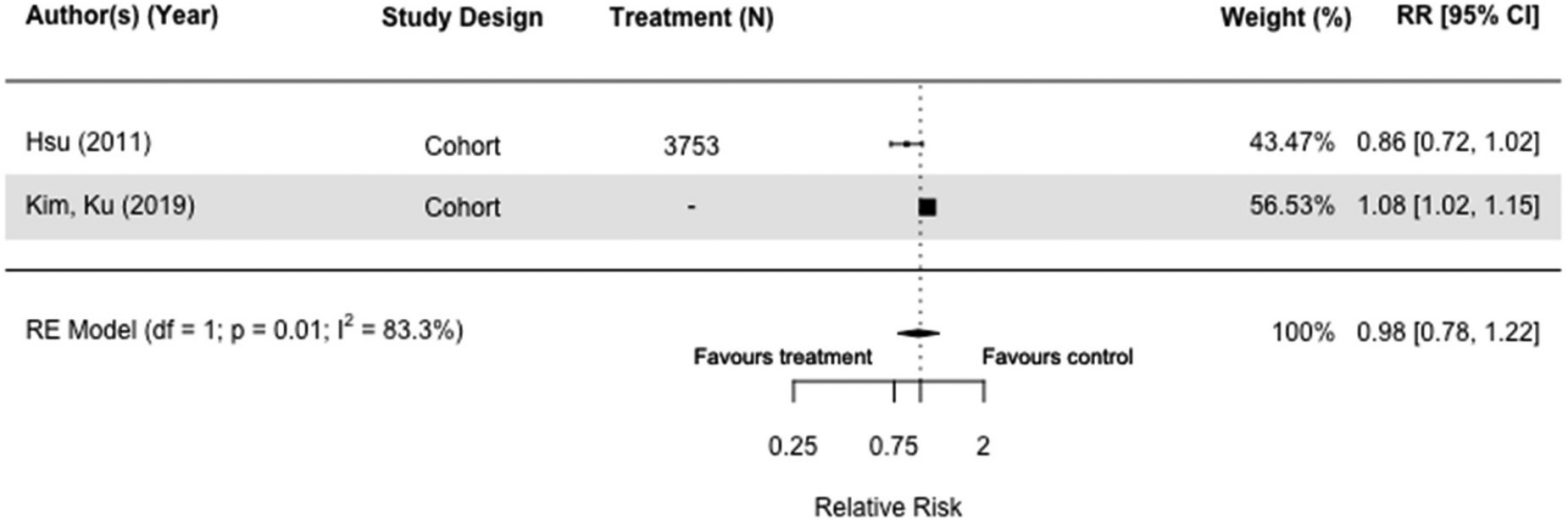
Forest plot of the effect of sulfonylureas (SU) versus those not taking SU (but on no diabetes medication(s)) on all-cause dementia in cohort studies.

Nine studies (N = 1,951,736) included sulfonylureas as an intervention. Of the three case-control^30,32,36^ and six cohort studies,^34,54,55,72^ five were meta-analyzed with the same comparators.^30,36,45,54,55^ Sulfonylureas did not impact dementia risk when we compared users with those taking other medication(s) in case-control studies (RR = 1.03[0.99, 1.07], I^2 ^= 0%) (Figure 4),^30,36^ and those who did not receive any diabetes medication (RR = 0.98[0.78, 1.22], I^2 ^= 83.3%) in cohort studies (Figure 5).^54,55^ Results of sensitivity analyses comparing users of sulfonylureas with those on any other or no medication(s) were also not significant (RR = 1.05[0.99, 1.11], I^2 ^= 79.6%) (Supplemental Figure 5),^30,36,54,55^ and case-control and cohort studies that included both all-cause dementia and Alzheimer's Dementia as an outcome mirrored the main findings (RR = 1.02[0.96, 1.09], I^2 ^= 82.8%) (Supplemental Figure 6).^30,32,36,45,54,55,66^

### Metformin

**Figure 6.**
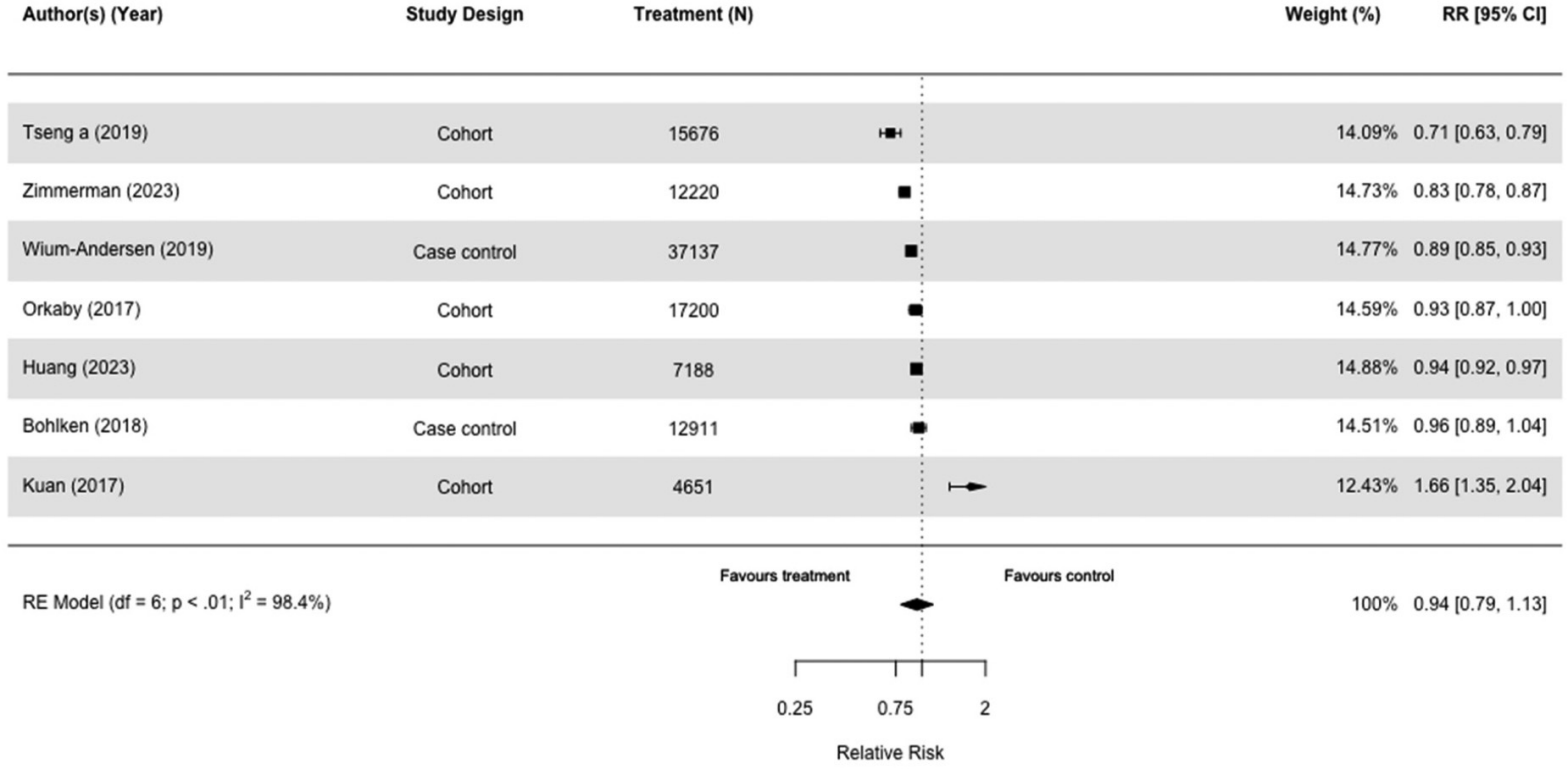
Forest plot of the effect of metformin versus those not taking metformin (but on other diabetes medication(s)) on all-cause dementia in case-control and cohort studies.

**Figure 7.**
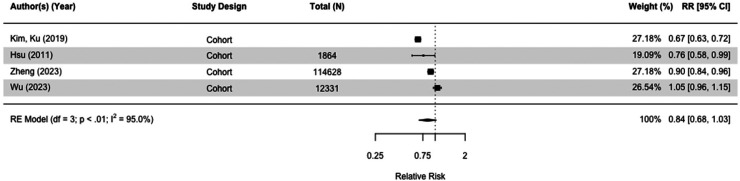
Forest plot of the effect to metformin versus those not taking metformin (but on no diabetes medication) on all-cause dementia in cohort studies.

Thirty-one studies, including 3,284,828 participants, assessed the impact of metformin (Tables 2 and 3; Supplemental Tables 2 and 3). When we included the seven studies comparing risk of all-cause dementia in metformin users versus those taking other diabetes medication(s), there was no difference (RR = 0.94[0.79, 1.13], I^2 ^= 98.4%) in case-control and cohort studies (Figure 6).^30,36,51,52,58,64,67^ Results were similar when we compared metformin users with those on no diabetes medication (RR = 0.84[0.68,1.03], I^2 ^= 95.0%) (Figure 7). We conducted multiple sensitivity analyses, and there was no overall difference. Emulated trials and cohort studies did not indicate metformin had a protective effect (Supplemental Figures 8–10, 12), but case controls studies found a small effect (RR = 0.92[0.85, 0.99], I^2 ^= 67.7%) (Supplemental Figure 1^1^). We meta-analyzed 15 studies, 10 cohort studies,^38,49,51,52,54,55,58,64,66,67^ and five case-control studies^30,32,35,36,53^ either taking no medication or other medication group with the outcome of dementia or Alzheimer's disease and found that the overall risk was 0.95 (95% CI = 0.84 to 1.08 with wide confidence intervals) of which two cohort studies were emulated trials (RR = 0.95[0.84, 1.08], I^2 ^= 97.8%) (Supplemental Figure 9).^51,58^

**Figure 8.**
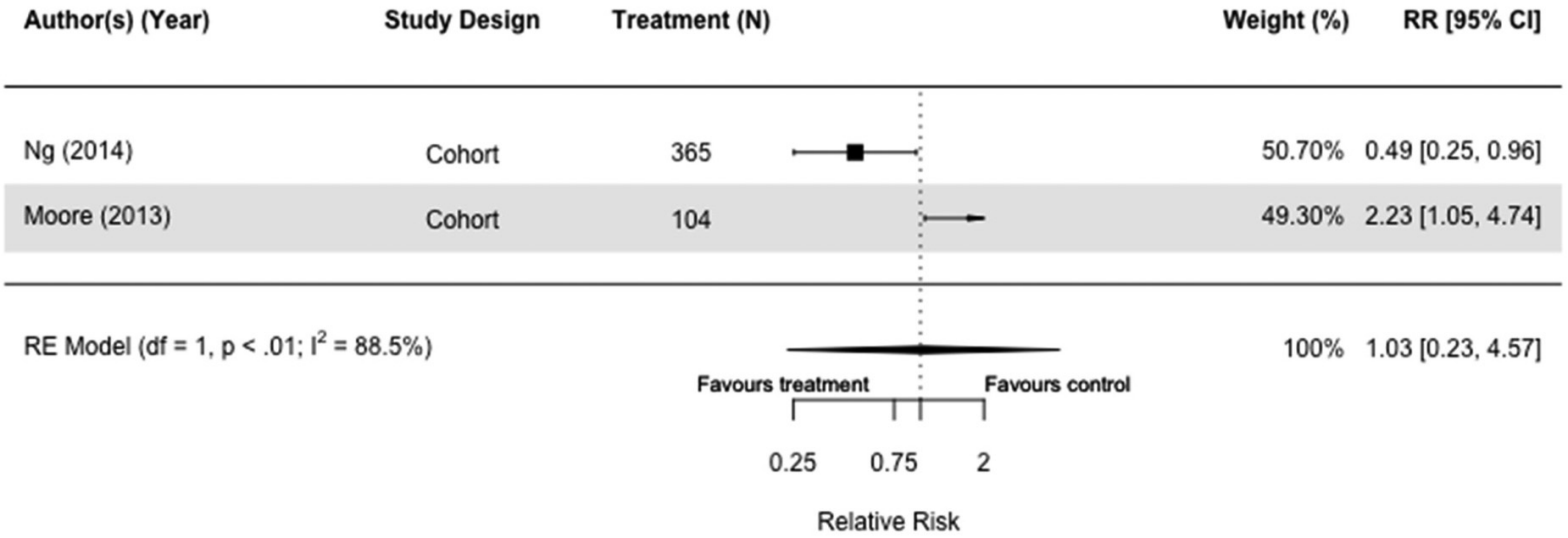
Forest plot of the effect to metformin on cognitive decline versus those not taking metformin (but on other or no diabetes medication(s)) in cohort studies.

Two cohort studies had cognitive decline as an outcome,^61,69^ and metformin did not impact cognitive decline (RR = 1.03[0.23, 4.57]. I^2 ^= 88.5%) (Figure 8).

### Insulin

**Figure 9.**
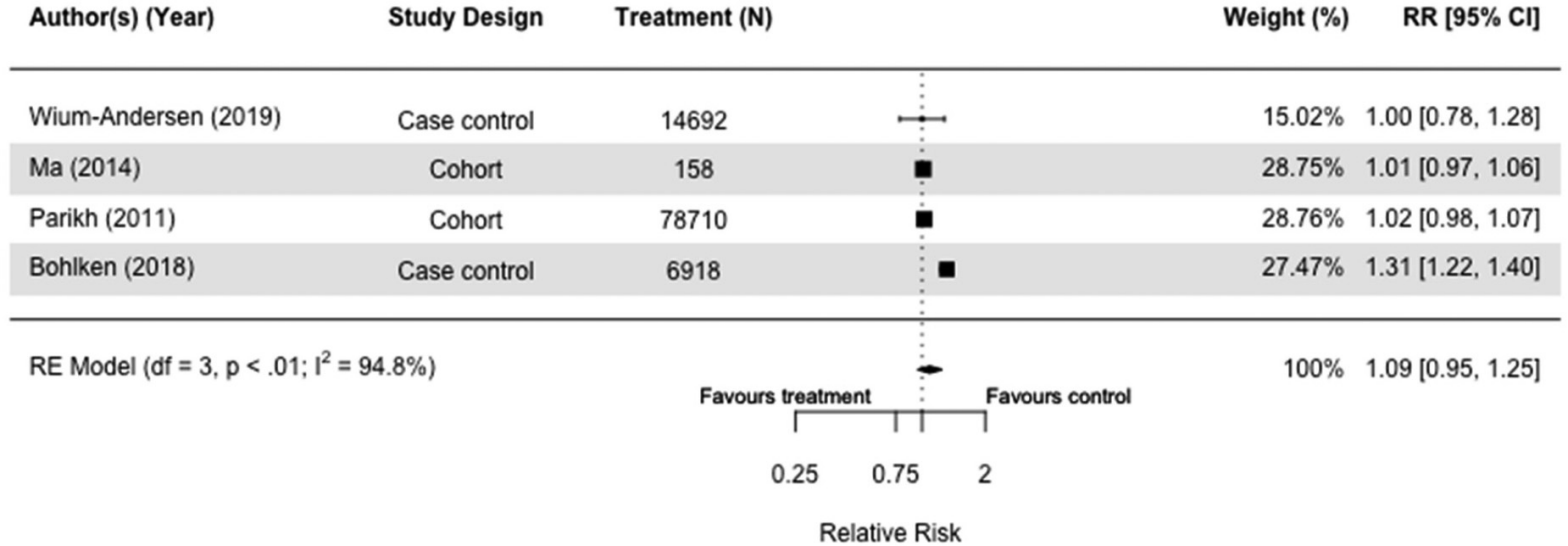
Forest plot of the effect of insulin versus those not taking insulin (but on other or no diabetes medication(s)) on all-cause dementia in case-control and cohort studies.

Of the nine studies, six cohort,^39,40,47,60,75^ and three case-control studies included insulin as treatment, resulting in a total of 629,006 participants.^30,32,36^ Six studies were meta-analyzed because they had the same comparison group.^30,32,36,40,60,66^ Insulin did not seem to affect dementia risk with wide parameters and high heterogeneity when users were compared to those taking other or no diabetes medication(s) (RR = 1.09[0.95, 1.25], I^2 ^= 94.8%) in case-control and cohort studies (Figure 9).^30,36,40,60^ When we included both all-cause dementia and Alzheimer's dementia as outcomes, the results were similar (RR = 1.06[0.91, 1.24], I^2 ^= 95.4%) (Supplemental Figure 1^3^).^30,32,36,40,60,66^

### Dipeptidyl peptidase iv inhibitors (DPP-4i)

**Figure 10.**
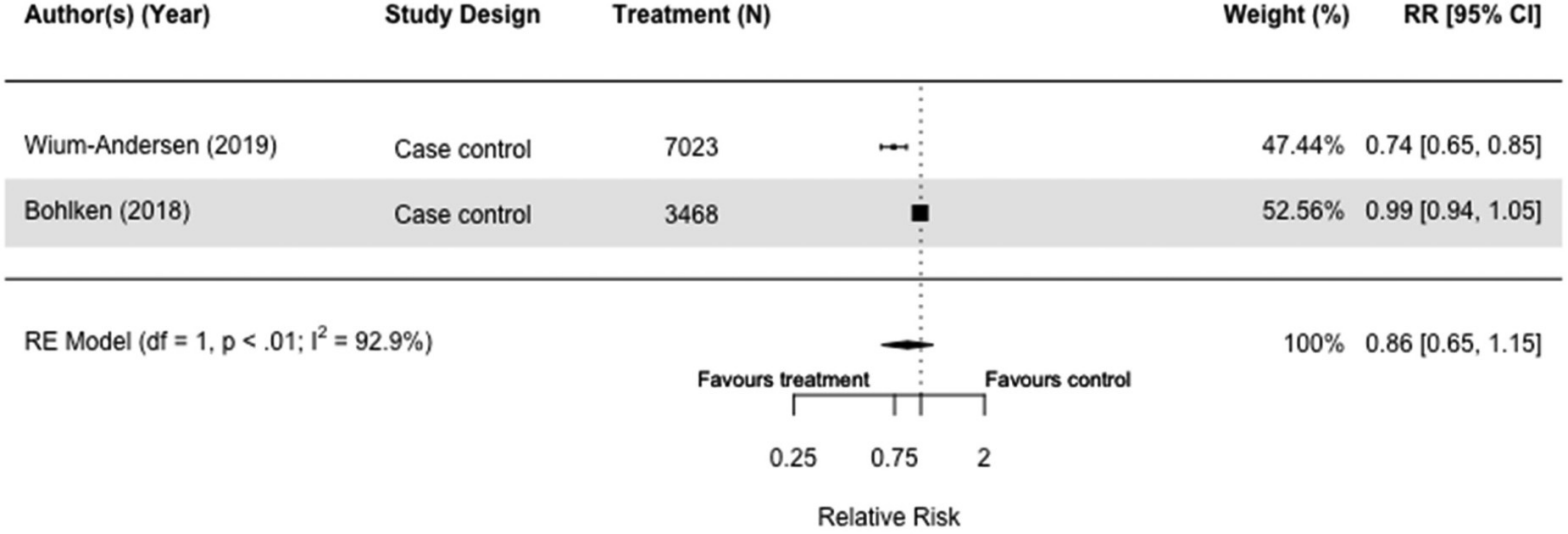
Forest plot of the effect of dipeptidyl peptidase IV inhibitors (DPP-4i) versus those not taking DPP-4i (but on other or no diabetes medication(s)) on all-cause dementia in case-control studies.

Of the six studies (N = 1,972,157) that included DPP-4i as a treatment, we meta-analyzed two case-control studies as they both compared users with non-users of DPP-4i. There was no significant association between the use of DPP-4i and reduced dementia risk. In addition, heterogeneity was high, and confidence intervals were wide (RR = 0.86[0.65, 1.15], I^2 ^= 92.9%) (Figure 10).^30,36^

## Discussion

This systematic review evaluates the impact of medications for diabetes on the development of dementia, MCI, or cognitive decline. Most studies were observational, but there were RCTs of two drugs. Three GLP-1 RA RCTs found that people taking it versus those on second-line diabetes medications for five years a 53% decrease risk of developing dementia, and a reduced risk of cognitive impairment.^33^ Findings from our meta-analysis of case-control studies with 5–17 years of follow-up showed an associated reduced risk of 27%. Similarly, two case-control studies controlled for diabetes duration and found an association between GLP-1 RA and reduced dementia risk of 19%.^30,33^

An RCT of repaglinide found that those on it had less cognitive decline over 12 months than those on glibenclamide. However, the changes in mean differences were small—0.8 points on the Mini-Mental State Examination (MMSE), and PwD decline by 3.3 points per annum on average.^76^ Abbatecola et al.^29^ proposed that there was less glycemic control in glibenclamide users, and that postprandial plasma glucose has potential impact on cognitive decline.^29^

Several drugs examined the effect of diabetes medication in observational studies. Our meta-analysis of seven studies found that glitazones consistently reduced the development of dementia by 22% in users versus those not taking glitazones but on other or no diabetes medication(s) in a range of 2–17 years. Due to low heterogeneity, it could be applied to other populations. Only two included studies adjusted for diabetes duration, of which only Bohlken et al.^30^ compared glitazones users to non-users, and found that glitazones were associated with an alleviated risk of dementia (19%).

We did not find an association between metformin and a reduction in the development of all-cause dementia or cognitive decline. Insulin did not appear to influence the development of dementia, MCI, or cognitive decline in the two-case-control and two cohort studies we meta-analyzed, with the follow-up duration of 5 to 17 years. This finding was the same in the two cohort studies that adjusted for diabetes duration, where one had MCI as an outcome and other had dementia.^47,74^

### Interpretation of the findings

Previous systematic reviews have considered the impact of the medications included in our meta-analysis. The two systematic reviews that evaluated GLP-1 RAs had conflicting findings. While Tian et al.^17^ found GLP-1 RAs to decrease dementia risk by 64.5%, Zhou et al.^15^ found no difference. Our findings for glitazones are in line with previous systematic reviews, suggesting that it is associated with reduced risk of dementia.^15,17^

In our review, we did not find that overall metformin or insulin impacted subsequent dementia but there was large heterogeneity. No RCTs fulfilled our inclusion criteria. Neither emulated trials nor cohort studies found a protective effect. However, when we conducted a meta-analysis of two case-control studies, metformin use was associated with an 8% reduction in dementia risk. Previously findings for metformin in the literature have been inconclusive but are strengthened by the negative results of emulated trials. Two past systematic reviews and meta-analyses have suggested metformin decreased dementia risk in people with type 2 diabetes.^77,78^ Other systematic reviews have reported no effect.^17,79,80^ More recently, Dai et al. evaluated time-related biases in studies that evaluated metformin and subsequent dementia, and they found that studies that addressed the most biases concluded no association between metformin and dementia risk.^80^ This is critical as diabetes duration might be a confounder. Previous animal studies have, for example, shown that metformin protects against deposition of plaques and tangles in mice.^81^ A Mendelian randomization study considering genetic proxies for metformin targets found that metformin might reduce risk of Alzheimer's disease through mitochondrial function.^82^ There are on-going trials evaluating the effect of metformin on healthy older adults for dementia prevention.^83,84^

Past reviews have indicated insulin to be associated with an increased risk of dementia. While our findings were non-significant and heterogeneous, it was the same direction.^15,16^ As with metformin, the finding might be influenced by diabetes duration and severity, which was not accounted for in most studies. It is paramount to adjust for diabetes duration as the longer people have had diabetes, the more severe it is likely to be, the higher the dementia risk,^85^ and the higher the chance they are on subcutaneous treatments, like insulin.^86^ Conversely, people with diabetes who are adherent to medication may have more controlled blood sugar and be less likely to develop cognitive disorders.^68^

### Potential mechanisms

GLP-1 RAs are considered one of the most promising drugs for repurposing as a treatment for dementia.^87^ We found the most robust evidence for reducing dementia risk with both RCTs and observational studies finding them protective, including those adjusting for duration of diabetes. Potential mechanisms include the amelioration of dementia risk factors, such as glycated hemoglobin, reduced body weight, decreased systolic blood pressure, and reduced the risk of cardiovascular disease.^33^ GLP-1 RAs role in reducing neuroinflammation may also be critical. Previous studies have found semaglutide to specifically decrease marks of inflammation in humans,^88,89^ and both liraglutide and semaglutide use an anti-inflammatory mechanism to attenuate development of atherosclerotic plaques in apoprotein E deficient (Apoe-/-) and low-density lipoprotein receptor deficient (LDLr -/-) mice.^90^ In addition to its glucose-lowering effects, GLP-1 RAs might have anti-dementia properties as a preliminary study showed liraglutide improved cerebral metabolism in mild to moderate dementia, and a recent study demonstrated that liraglutide slowed down the decline of memory function independently of weight loss in a group of people with obesity and pre-diabetes or early type 2 diabetes.^91^

Glitazones are potent insulin-sensitizing drugs that act on intracellular metabolic pathways to enhance insulin action and sensitivity. While we included both rosiglitazone and pioglitazone in this review, rosiglitazone is banned in the UK due to its effect on raising the risk of heart disease.^92^ Although suspended in Europe, rosiglitazone is allowed in the US under tight restrictions.^93^ In addition to glycemic control, glitazones may exert neuroprotective effects by reducing levels of amyloid-β, inhibiting tau hyperphosphorylation and synaptic plasticity and restoring cerebral vascular function, reducing inflammation response by microglia and astrocytes.^94,95^

Obesity is a known risk factor for dementia,^23^ and the weight loss induced by metformin, or its underlying mechanisms—such as its ability to reduce insulin levels—suggests it may help lower the risk of dementia. Previous studies have shown that sulfonylureas are associated with weight gain due to increased insulin production,^96^ which could further contribute to insulin resistance.

### Strengths and limitations

Our findings, from numerous countries and studies with various study designs, provides the most up-to-date evidence. The systematic nature of this review and the meta-analysis allowed us to provide a comprehensive evaluation of the current diabetes medication on the development of dementia, MCI, or cognitive decline. While previous reviews have considered the impact of diabetes medication on dementia risk, all combined the diabetes medication in their meta-analyses, making it difficult to draw conclusions on the impact of each drug on dementia risk independently.^15–17^ One of the strengths of this study is we considered each treatment individually with non-users of the same drug or no medication.

Since most of our included studies are observational, and only six cohort studies controlled for^30,35,47,48,61,74^ and 3 case-control studies matched for diabetes duration,^33,53,59^ and four adjusted for diabetes severity,^39,45,58,59^ there is the possibility of confounding by indication. The lack of evidence for a treatment also does not mean lack of efficacy. We did not present information on dosage and adherence as only 13 studies included information on dosage and none on adherence.^30,36,45,51,54,56,58,59,63–65,67,68^ Another limitation is we could not consider the source of heterogeneity, because of the few studies in each meta-analysis. While all the studies we meta-analyzed adjusted for age and sex, not all adjustments were the same. Some adjusted for additional factors, such as socioeconomic status, which increases between-study variation and adds to heterogeneity.

### Implication of findings/conclusion

Overall, there is considerable evidence suggesting that taking diabetes medication for people who have diabetes could potentially reduce the risk of dementia, MCI, or cognitive decline. However, only GLP-1 RAs have RCT evidence, and observational studies are limited by confounding by indication. For example, those who take medication could differ systematically from those who do not.

Future observational studies could consider adjusting for diabetes severity and duration. RCTs are unlikely to provide long-term outcomes. Glitazones could be further studied as they had homogenous results. A recent Cochrane review proposed that there is no or very little difference between RCTs and observational studies when heterogeneity is low (<50%).^97^ While we do not have RCT evidence for glitazones, our meta-analyses I^2^ were consistently 0%, even in sensitivity analyses where we included studies with Alzheimer's dementia as an outcome.^97^ Studies could also investigate on what dose and length of drug use of GLP-1 RAs would be ideal in reducing the risk of dementia.

## Supplemental Material

sj-docx-1-alz-10.1177_13872877251319054 - Supplemental material for Effect of diabetes medications on the risk of developing dementia, mild cognitive impairment, or cognitive decline: A systematic review and meta-analysis Supplemental material, sj-docx-1-alz-10.1177_13872877251319054 for Effect of diabetes medications on the risk of developing dementia, mild cognitive impairment, or cognitive decline: A systematic review and meta-analysis by Esther K. Hui, Naaheed Mukadam, Gianna Kohl and Gill Livingston in Journal of Alzheimer's Disease

## Data availability

Data supporting the findings of this study are available on request from the corresponding author.
