# Supplementary material for: Effect of diabetes medications on the risk of developing dementia, mild cognitive impairment, or cognitive decline: A systematic review and meta-analysis
Source: J Alzheimers Dis. 2025 Feb 27;104(3):627–48. doi: 10.1177/13872877251319054 (PMC12231844; doi:10.1177/13872877251319054)
Supplement: sj-docx-1-alz-10.1177_13872877251319054 - Supplemental material for Effect of diabetes medications on the risk of developing dementia, mild cognitive impairment, or cognitive decline: A systematic review and meta-analysis [file sj-docx-1-alz-10.1177_13872877251319054.docx]

**Supplemental Material**

**Effect of diabetes medications on the risk of developing dementia, mild cognitive impairment, or cognitive decline: A systematic review and meta-analysis**

**
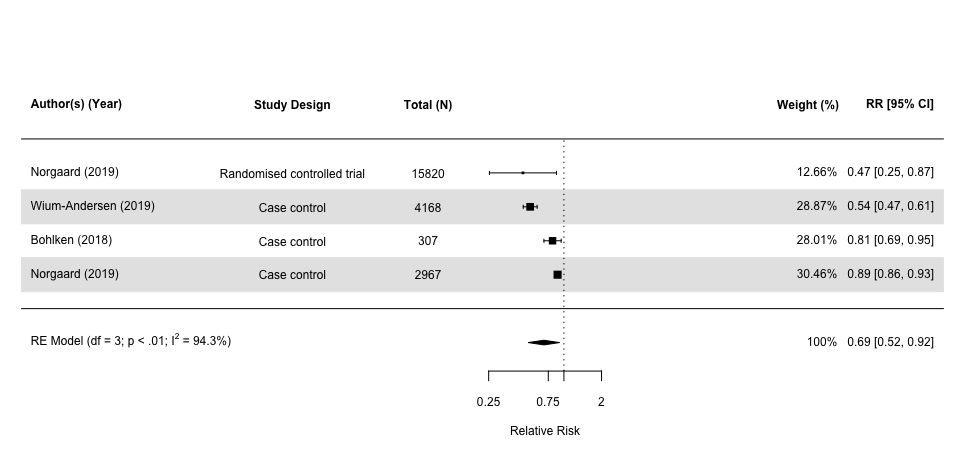
**

**Supplemental Figure 1.** Forest plot of the effect of glucagon-like peptide 1 receptor agonists (GLP-1 RA) versus those not taking GLP-1 RA (but on other or no diabetes medication(s)) on all-cause dementia in case-control studies and randomized controlled trials.


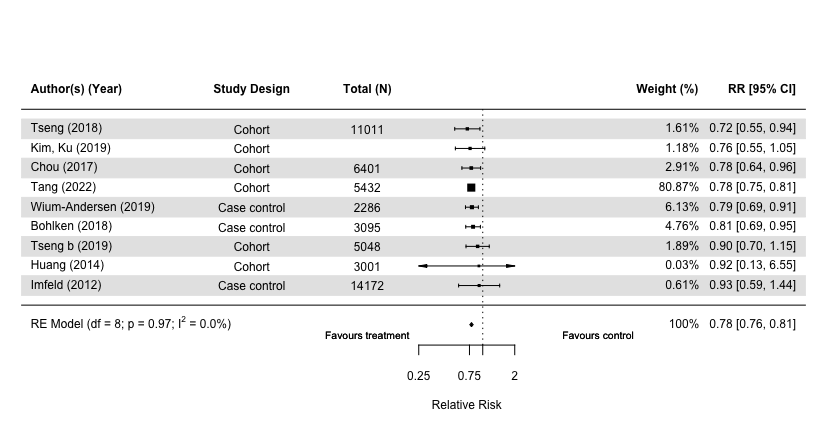


**Supplemental Figure 2.** Forest plot of the effect of glitazones verses those not taking glitazones (but on other or no diabetes medication(s)) on all-cause dementia or Alzheimer’s disease in case-control and cohort studies.


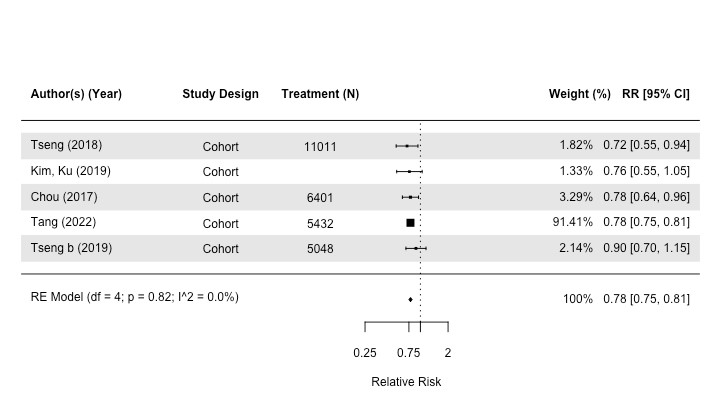


**Supplemental Figure 3.** Forest plot of the effect of glitazones verses those not taking (but on other or no diabetes medication) on all-cause dementia in cohort studies.


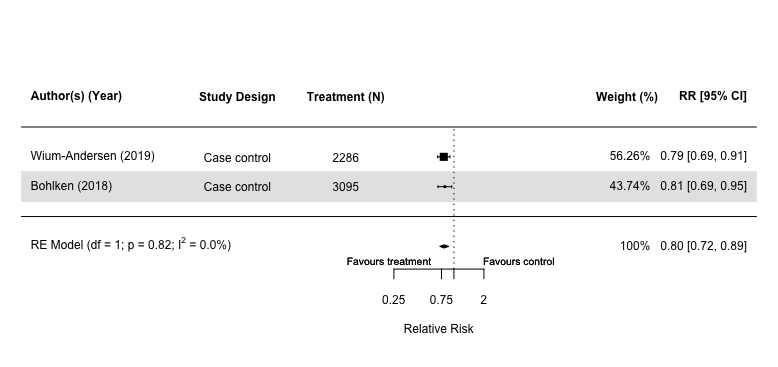


**Supplemental Figure 4.** Forest plot of the effect of glitazones versus those not taking glitazones (but on other or no diabetes medication) on all-cause dementia in case-control studies.


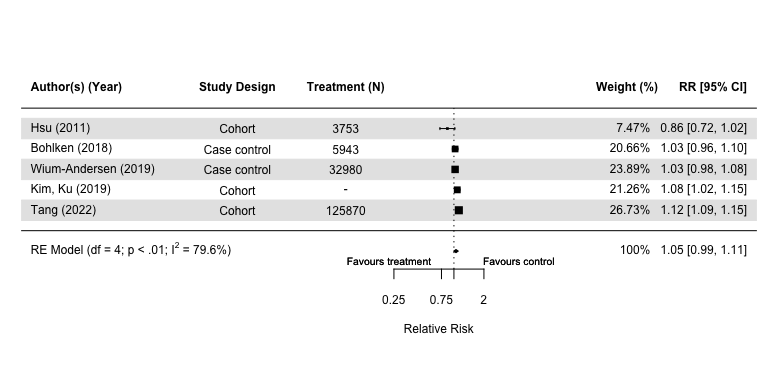


**Supplemental Figure 5.** Forest plot of the effect of sulfonylureas (SU) versus those not taking SU (but on other or no diabetes medication(s)) on all-cause dementia in case-control and cohort studies.


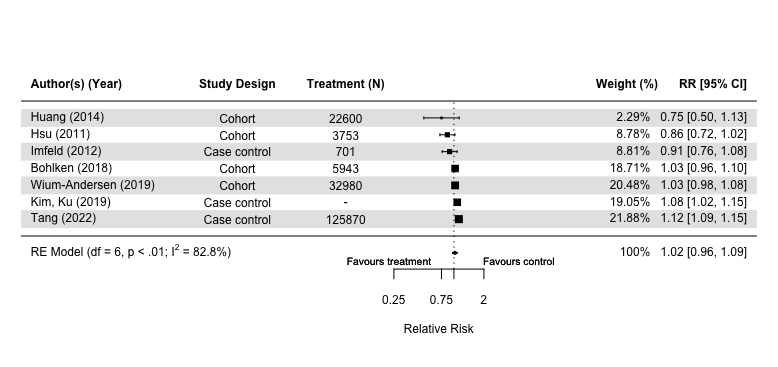


**Supplemental Figure 6.** Forest plot of the effect of sulfonylureas (SU) versus those not taking SU (but on other or no diabetes medication(s)) on all-cause dementia or Alzheimer’s disease in case-control and cohort studies.


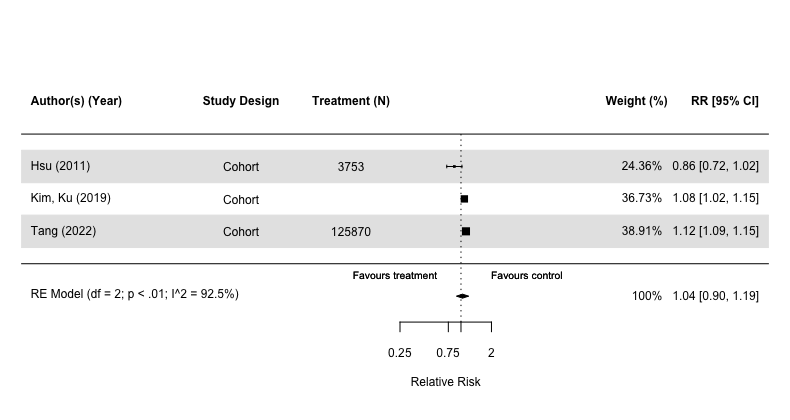


**Supplemental Figure 7.** Forest plot of the effect of sulfonylureas (SU) versus those not taking SU (but on other or no diabetes medication(s)) on all-cause dementia in cohort studies.


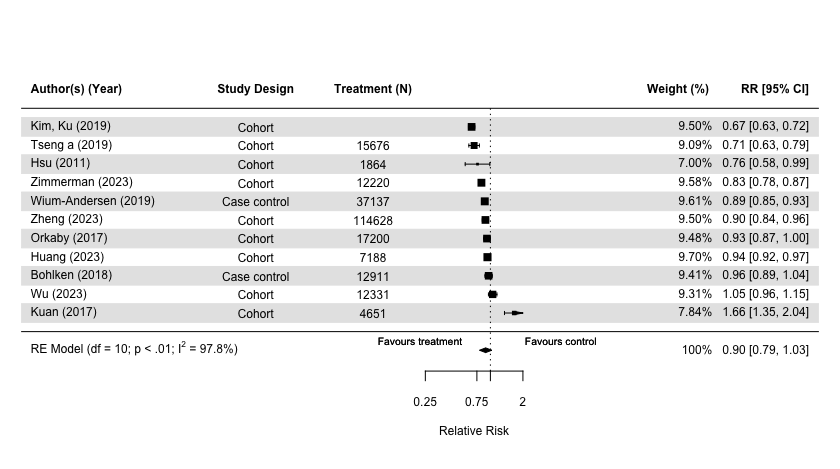


**Supplemental Figure 8.** Forest plot of the effect of metformin versus those not taking metformin (but on other or no diabetes medication(s)) on all-cause dementia in case-control and cohort studies.


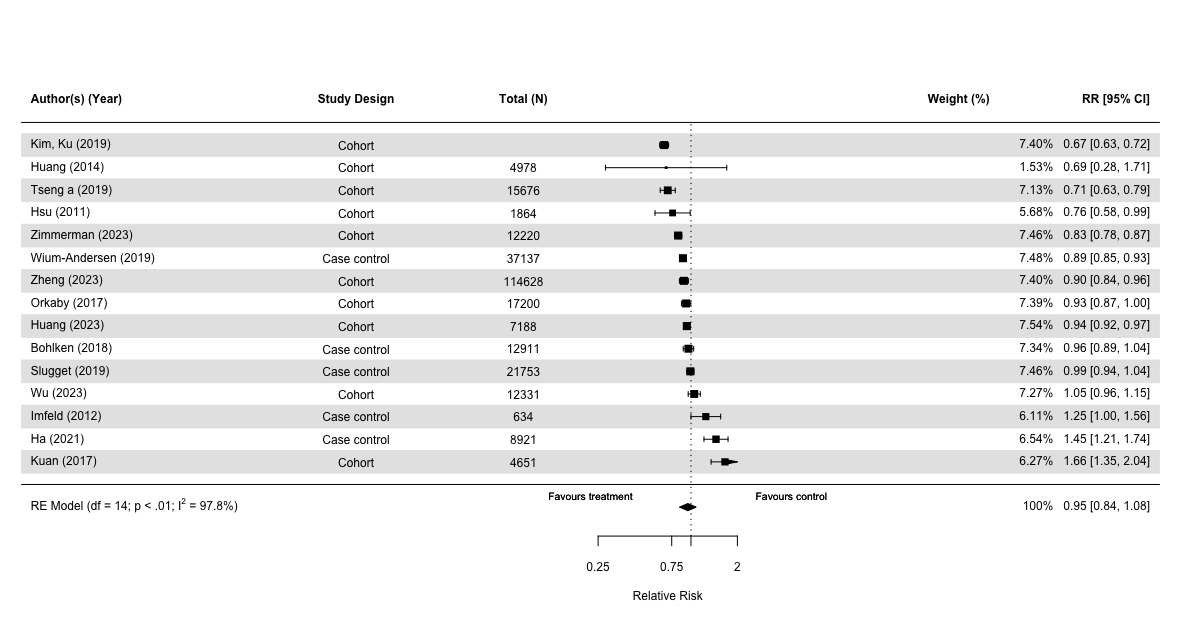


**Supplemental Figure 9.** Forest plot of the effect of metformin versus those not taking metformin (but on other or no diabetes medication(s)) on all-cause dementia or Alzheimer’s disease in case-control and cohort studies.


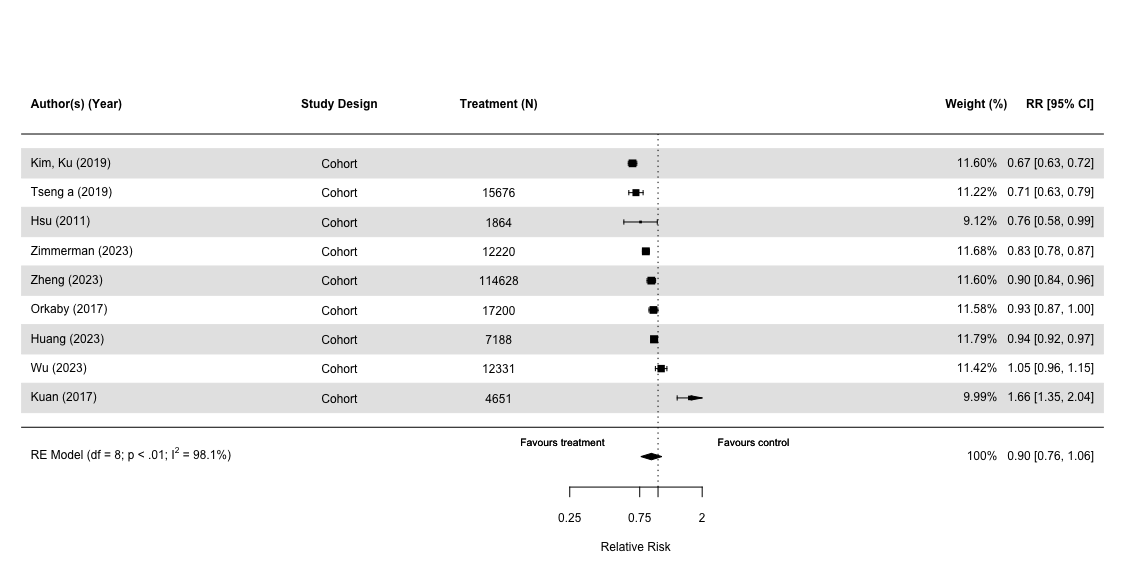


**Supplemental Figure 10.** Forest plot of the effect of metformin versus those not taking metformin (but on other or no diabetes medication(s)) on all-cause dementia in cohort studies.


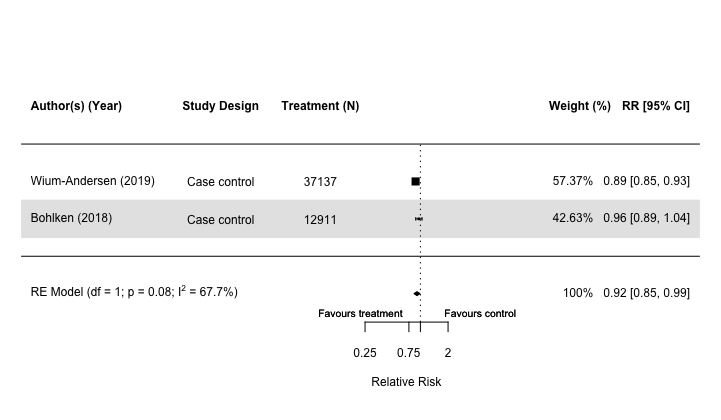


**Supplemental Figure 11.** Forest plot of the effect of metformin versus those not taking metformin (but on other diabetes medication(s)) on all-cause dementia in case-control studies.


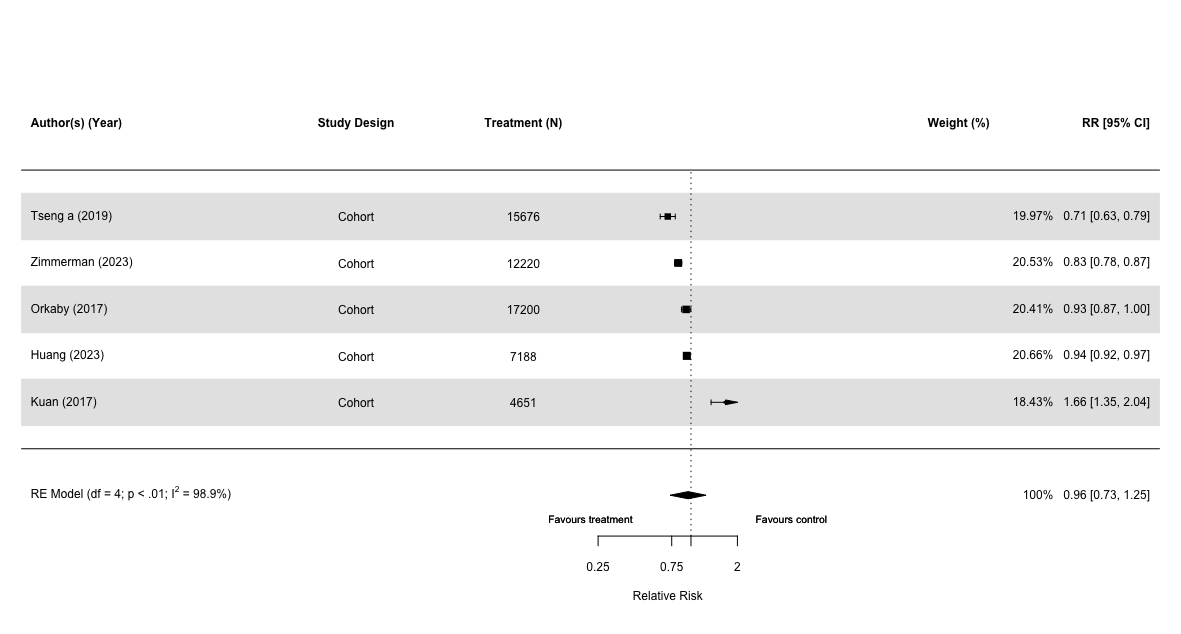


**Supplemental Figure 12.** Forest plot of the effect of metformin versus those not taking metformin (but on other diabetes medication(s)) on all-cause dementia in cohort studies.


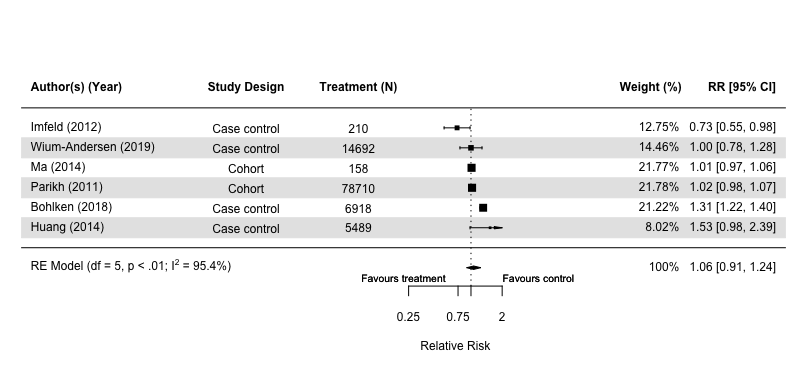


**Supplemental Figure 13.** Forest plot of the effect of insulin on those not taking insulin (but on other or no diabetes medication(s)) on all-cause dementia or Alzheimer’s disease in case-control and cohort studies.

**Sensitivity analysis**


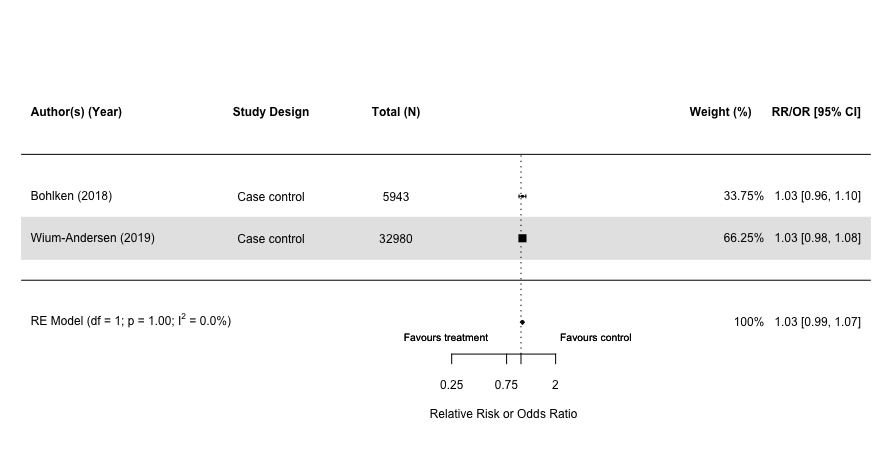


**Supplemental Figure 14.** Forest plot of the effect of sulfonylureas (SU) versus those not taking SU (but on other or no diabetes medication(s)) on all-cause dementia in case-control studies.


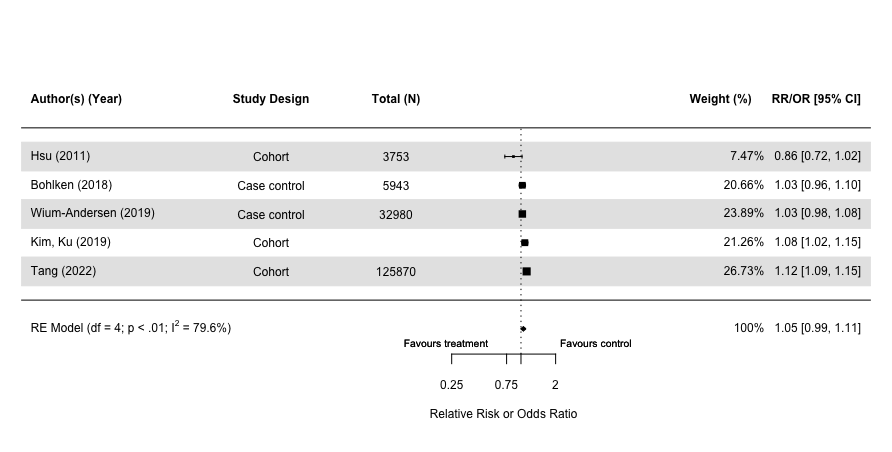


**Supplemental Figure 15.**  Forest plot of the effect of sulfonylureas (SU) on versus those not taking SU (but on other or no diabetes medication(s)) on all-cause dementia in case-control and cohort studies.


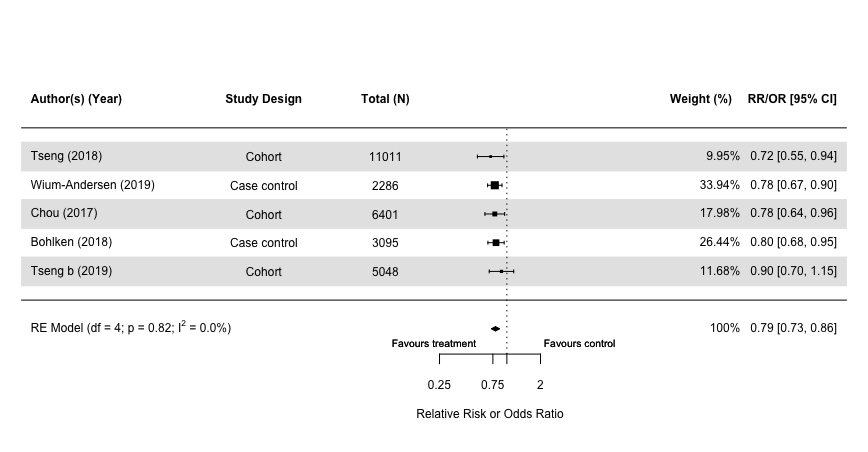


**Supplemental Figure 16.** Forest plot of the effect of glitazones versus those not taking glitazones (but on other or no diabetes medication(s)) on all-cause dementia in case-control and cohort studies.


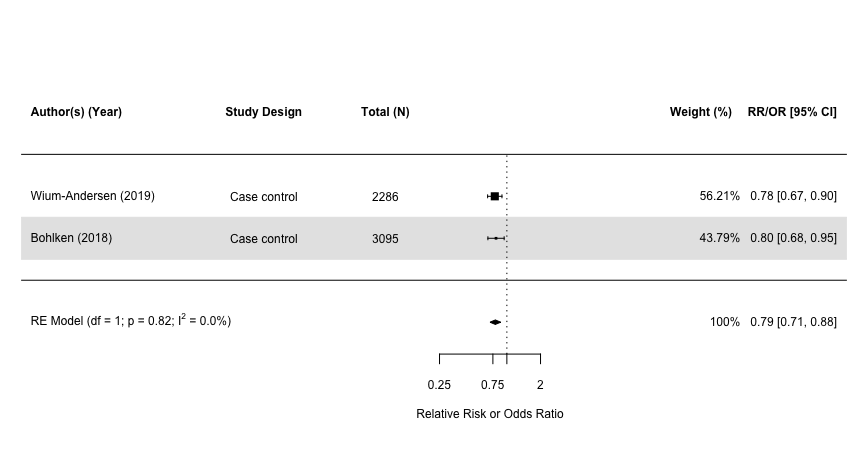


**Supplemental Figure 17.** Forest plot of the effect of glitazones versus those not taking glitazones (but on other or no diabetes medication(s)) on all-cause dementia in case-control studies.


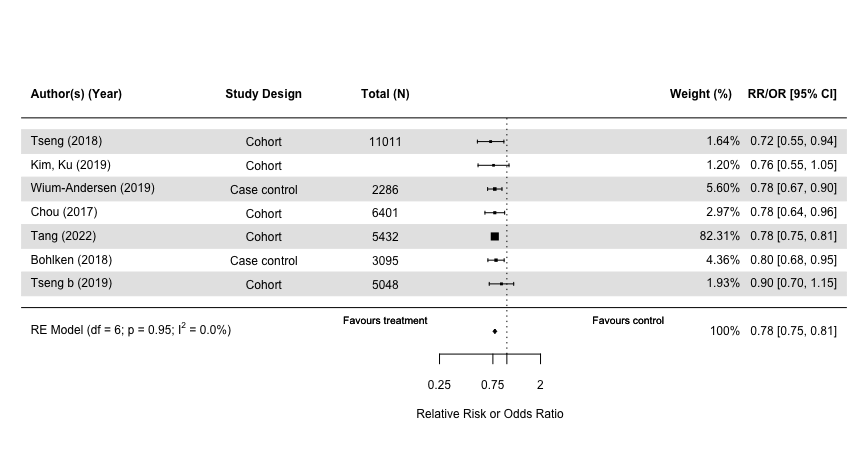


**Supplemental Figure 18.** Forest plot of the effect of glitazones versus those not taking glitazones (but on other or no diabetes medication(s)) on all-cause dementia in case-control and cohort studies.


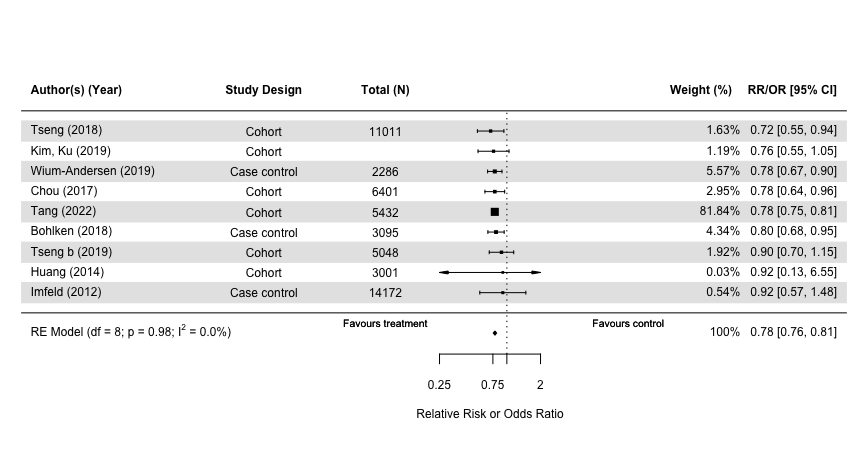


**Supplemental Figure 19.** Forest plot of the effect of glitazones versus those not taking glitazones (but on other or no diabetes medication(s)) on all-cause dementia or Alzheimer’s disease in case-control and cohort studies.


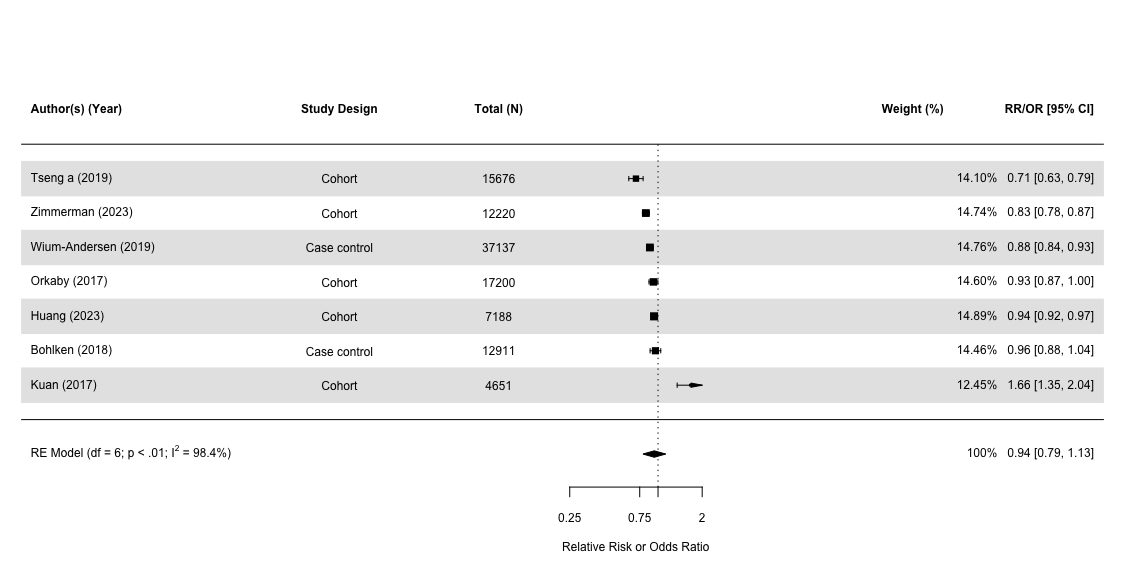


**Supplemental Figure 20.** Forest plot of the effect of metformin versus those not taking metformin (but on other or no diabetes medication(s)) on all-cause dementia in case-control and cohort studies.


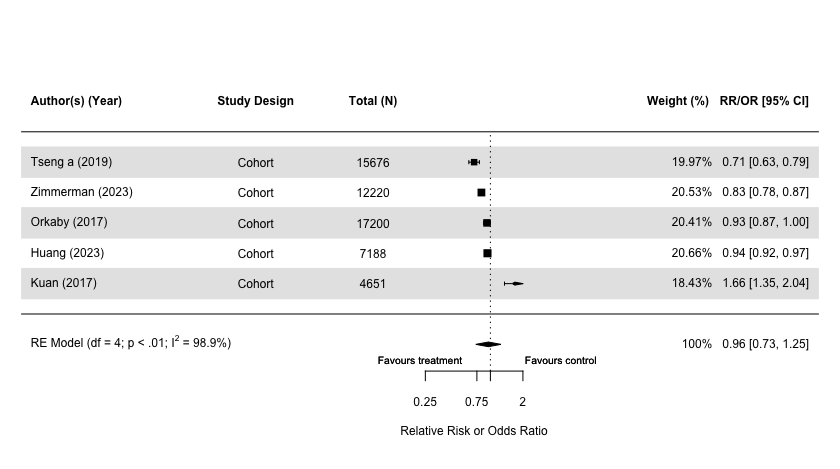


**Supplemental Figure 21.** Forest plot of the effect of metformin versus those not taking metformin (but on other or no diabetes medication(s)) on all-cause dementia in cohort studies.


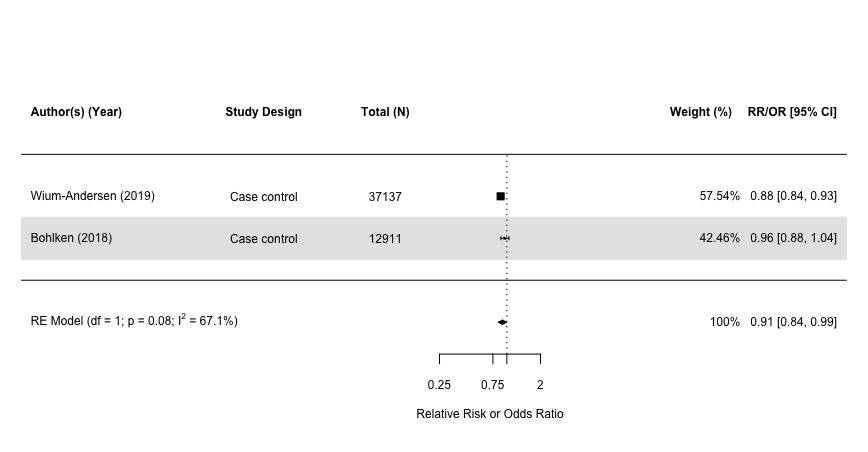


**Supplemental Figure 22.** Forest plot of the effect to metformin versus those not taking metformin (but on other or no diabetes medication(s)) on all-cause dementia in case-control studies.


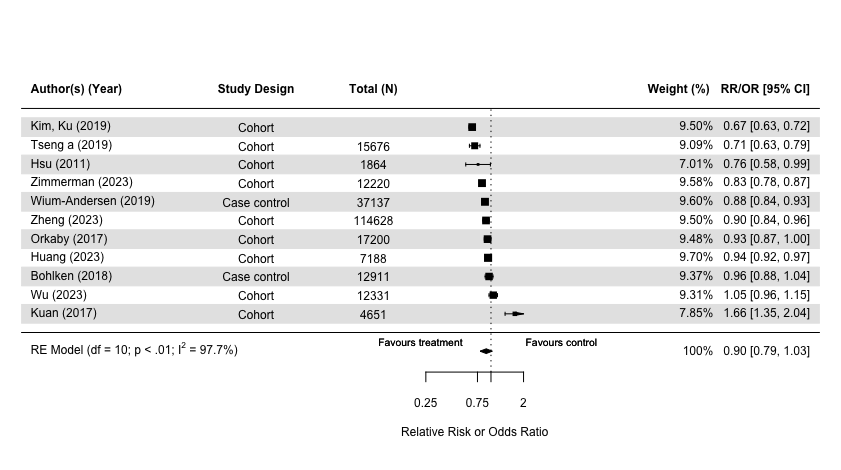


**Supplemental Figure 23.** Forest plot of the effect to metformin on versus those not taking metformin (but on other or no diabetes medication(s)) on all-cause dementia in case-control and cohort studies.


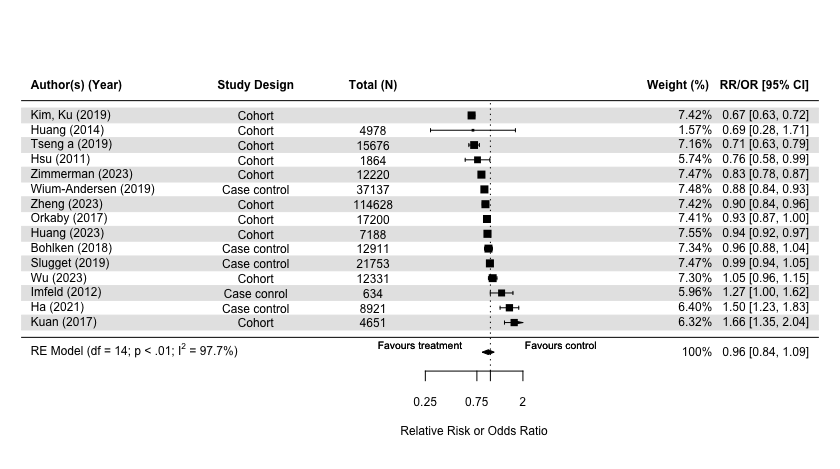


**Supplemental Figure 24.** Forest plot of the effect to metformin versus those not taking metformin (but on other or no diabetes medication(s)) on all-cause dementia or Alzheimer’s disease in case-control and cohort studies.


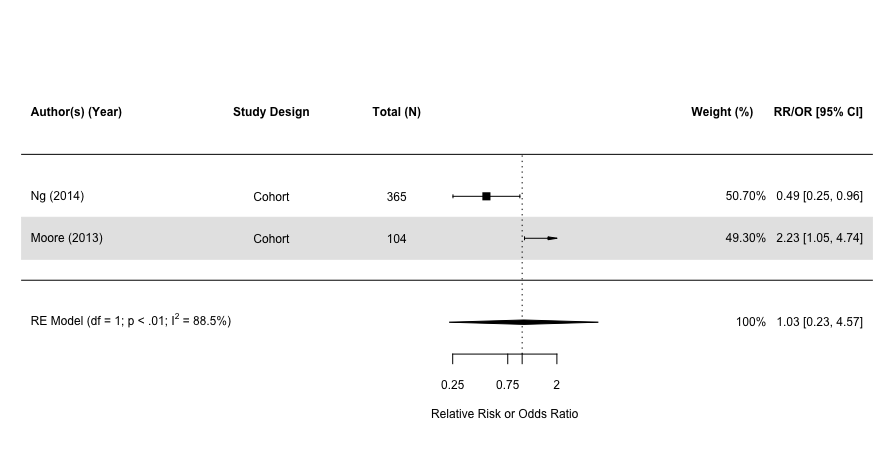


**Supplemental Figure 25.** Forest plot of the effect to metformin versus those not taking metformin (but on other or no diabetes medication(s)) on cognitive decline in cohort studies.


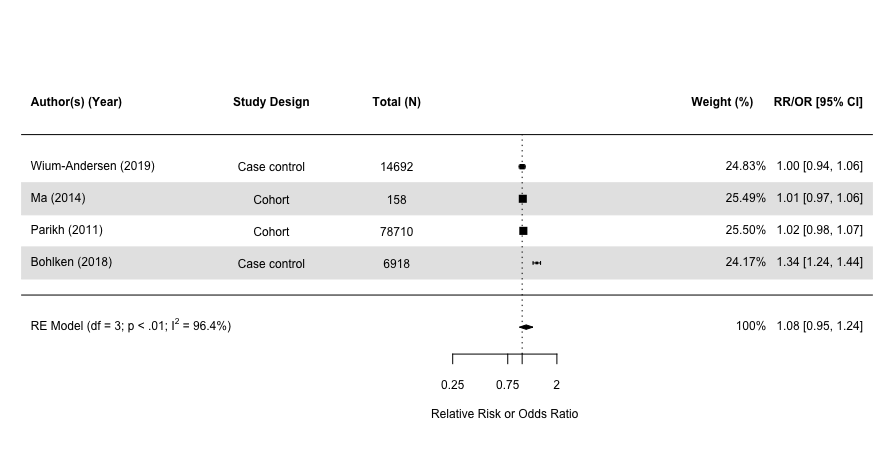


**Supplemental Figure 26.** Forest plot of the effect of insulin versus those not taking insulin (but on other or no diabetes medication(s)) on all-cause dementia in case-control and cohort studies.


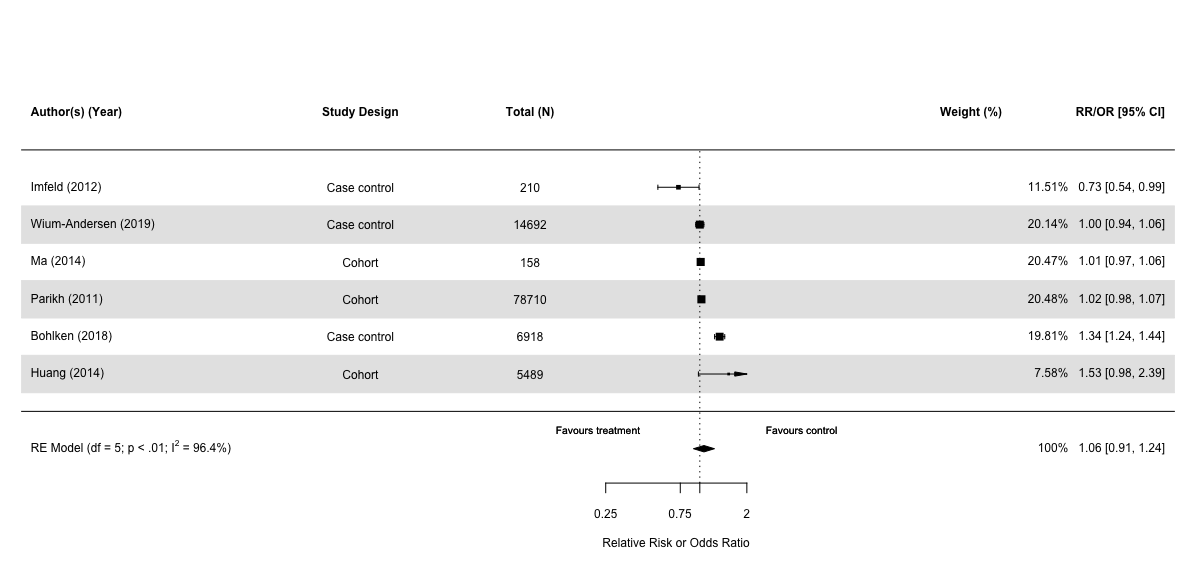


**Supplemental Figure 27.** Forest plot of the effect of insulin versus those not taking insulin (but on other or no diabetes medication(s)) on all-cause dementia or Alzheimer’s disease in case-control and cohort studies.


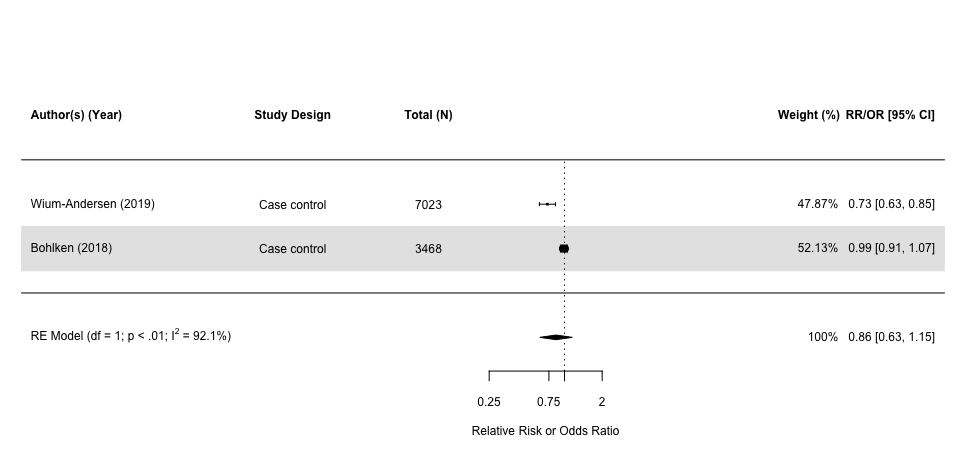


**Supplemental Figure 28.** Forest plot of the effect of dipeptidyl peptidase IV inhibitors (DPP-4i) versus those not taking DPP-4i (but on other or no diabetes medication(s)) on all-cause dementia in case-control studies.


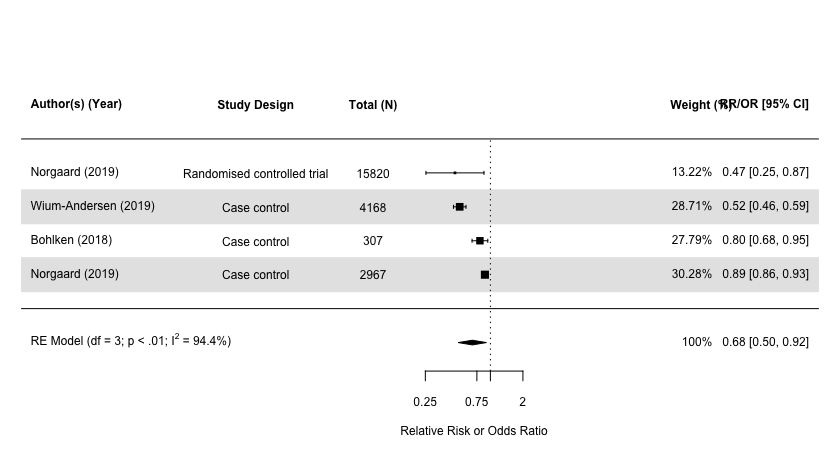


**Supplemental Figure 29.** Forest plot of the effect of glucagon-like peptide 1 receptor agonists (GLP-1 RA) versus those not taking GLP-1 RA (but on other or no diabetes medication(s)) on all-cause dementia in case-control studies and a randomized controlled trial.


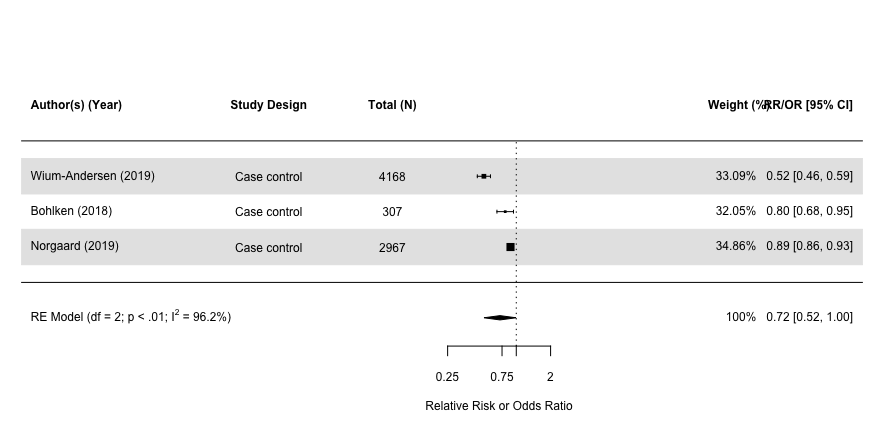


**Supplemental Figure 30.** Forest plot of the effect of glucagon-like peptide 1 receptor agonists (GLP-1 RA) versus those not taking GLP-1 RA (but on other or no diabetes medication(s)) on all-cause dementia in case-control studies.

**Supplemental Table 1.** Study characteristics of included randomized controlled trials (cognitive decline)

| **Study** | **Country** | **Sample size** | **Female (%)** | **Mean age (SD)** | **Mean/median/range follow-up years** | **Intervention** | **Comparator** | **Outcome** | **Outcome measure** | **Treatment effect (RR[95%CI]/mean∆)** | **Study quality** |
| --- | --- | --- | --- | --- | --- | --- | --- | --- | --- | --- | --- |
| Abbatecola (2006) | Italy | 156 | 48% | 74.40 (2.40) | Mean=1 year (time of follow-up) | Repaglinide | Gibenclamide | Cognitive decline | MMSE | Repaglinide: Mean∆= -1.3 | 8 |
|  |  |  |  |  |  |  |  |  |  | Glibenclamide: Mean∆= -0.5 |  |
| Cukiermann-Yaffe (2020) | Canada | 9,901 | 46.53% | 66 (6.4) | Median=5.4 years (IQR: 5.1-5.9) | Dulaglutide | Placebo (treatment as usual, can take up to two other non-metformin diabetes medication) | Cognitive decline | MoCA | Dulaglutide: RR=0.86 (0.79,0.95) | 10 |

CI: confidence intervals, DPP-4i: dipeptidyl peptidase-4 inhibitors, GLP-1 RA: glucagon-like peptide-1 receptor agonists, mean∆: change in pre-test and post-test scores, MMSE: Mini-Mental State Examination, MoCA: Montreal Cognitive Assessment, RR: relative risks, SD: standard deviation, IQR: interquartile range.

**Supplemental Table 2.** Study characteristics of included cohort studies (MCI, AD or cognitive decline)

| **Study** | **Country** | **Sample size** | **Female (%)** | **Intervention** | **Comparator** | **Mean age (SD)** | **Mean/median/ range follow-up years** | **Outcome** | **Treatment effect (RR[95%CI])** | **Study quality** |
| --- | --- | --- | --- | --- | --- | --- | --- | --- | --- | --- |
| Huang (2014)** | Taiwan | 71,433 | 48.00% | Metformin, SU, glitazones, α-glucosidase blockers, Non-SU insulin secretagogue, insulin | Non-use of all antidiabetic medication for monotherapy; non-use of the corresponding drug for combination therapy | 58.7 (14) | 5.5 years (mean) | AD | 1. Metformin: RR=0.69 (0.28,1.71) | 7 |
|  |  |  |  |  |  |  |  |  | 2. Metformin and oral antidiabetic medications: RR= 0.57 (0.26,1.26) |  |
|  |  |  |  |  |  |  |  |  | 3. SU: RR=0.75 (0.50,1.13) |  |
|  |  |  |  |  |  |  |  |  | 4. SU and oral antidiabetic medications: RR=0.59 (0.25,1.37) |  |
|  |  |  |  |  |  |  |  |  | 5. Glitazones: RR=0.92 (0.13,6.60) |  |
|  |  |  |  |  |  |  |  |  | 6. Glitazones and oral antidiabetic medications: RR=0.86 (0.36,2.02) |  |
|  |  |  |  |  |  |  |  |  | 7. α-glucosidase blockers: RR=0.71 (0.18,2.89) |  |
|  |  |  |  |  |  |  |  |  | 8. α-glucosidase blockers and oral antidiabetic medications: RR=1.37 (0.64,2.93) |  |
|  |  |  |  |  |  |  |  |  | 9. Non-SU insulin secretagogue: RR=1.33 (0.64,2.75) |  |
|  |  |  |  |  |  |  |  |  | 10. Non-SU insulin secretagogue and oral antidiabetic medication: RR=2.11 (0.93,4.77) |  |
|  |  |  |  |  |  |  |  |  | 11. Insulin: RR=1.53 (0.98,2.39) |  |
|  |  |  |  |  |  |  |  |  | 12. Insulin and oral antidiabetic medication: RR=2.17 (1.04,4.52) |  |
| Kuan (2017) | Taiwan | 9,302 | 49.52% | Metformin | Non-use of metformin | 64.7 (9.73) | 12 years (range) | AD | 2. Metformin (AD): RR=2.13 (1.20,3.79) | 8 |
| Moore (2013) | Australia | 1,354 | 59.50% | Metformin | Non-use of metformin | 73.86 (8.3) | Missing | Cognitive decline | 1. Metformin: RR=1.94 (1.05,3.82) | 5 |
| Ng (2014) | Singapore | 365 | 58.36% | Metformin | Non-use of metformin | 67.29 (7.37) | 4 years (mean) | Cognitive decline | 1. Metformin: RR=0.49 (0.25,0.95) | 5 |
| Orkaby (2017) | US | 17,200 | 1% | Metformin | Sulfonylureas | 74 (5.9) | 5 years (mean) | AD | 1. Age<75: RR=0.93 (0.76,1.14) 2. Age≥75: RR=0.95 (0.82,1.10) | 7 |
| Rizzo (2014) | Italy | 120 | 59.20% | DPP-4i, metformin, SU | DPP-4i/SU | 72.8 (4.35) | 2 years (mean) | Cognitive decline | Could not extract data from graph | 5 |
| Shi (2019) | US | 5,528 | 2% | Metformin | Non-use of metformin | 63.24 (10.85) | 6 years (range) | AD | 1. AD, ≤1 year: RR=2.19 (1.21,3.94) | 4 |
|  |  |  |  |  |  |  |  |  | 2. 1-2 years: RR=0.86 (0.33,2.21) |  |
|  |  |  |  |  |  |  |  |  | 3. 2-4 years: RR=0.63 (0.26,1.50) |  |
|  |  |  |  |  |  |  |  |  | 4. >4 years: RR=0.17 (0.04,0.70) |  |
|  |  |  |  |  |  |  |  |  |  |  |
|  |  |  |  |  |  |  |  |  | 1. MCI, ≤1 year: RR=0.86 (0.19,3.81) |  |
|  |  |  |  |  |  |  |  |  | 2. 1-2 years: RR=1.50 (0.36,6.19) |  |
|  |  |  |  |  |  |  |  |  | 3. 2-4 years: RR=1.43 (0.41,4.95) |  |
|  |  |  |  |  |  |  |  |  | 4. >4 years: RR=0.78 (0.19,3.19) |  |
| Wennberg (2018) | US | 508 | 40.50% | Metformin, oral medication, insulin | Non-use of corresponding drug | 74.5 (70.7,80.5)* | 3.6 years (median) | MCI | 1. Metformin: RR=2.75 (1.64,4.63) | 5 |
|  |  |  |  |  |  |  |  |  | 2. Other oral medication: RR=1.96 (1.19,3.25) |  |
|  |  |  |  |  |  |  |  |  | 3. Insulin: RR=3.17 (1.27,7.92) |  |
| Xu (2022) | UK | 495,942 | 54.54% | Glucosamine | Non-use of Glucosamine | 56.54 (8.09) | 11 years (median) | AD | 1. AD: RR=0.96 (0.86,1.07) | 6 |

AD: Alzheimer's disease, CI: confidence intervals, DPP-4i: dipeptidyl peptidase-4 inhibitors, MCI: mild cognitive impairment, RR: relative risks, SD: standard deviation, SU: sulfonylureas.

*median and interquartile range

**nested case-control study

**Supplemental Table 3.** Study characteristics of included case-control studies (AD)

| **Study** | **Country** | **Sample size** | **Study design** | **Female (%)** | **Mean age (SD)** | **Mean/range follow-up years** | **Intervention** | **Comparator** | **Treatment effect (RR[95%CI])** | **Study quality** |
| --- | --- | --- | --- | --- | --- | --- | --- | --- | --- | --- |
| Ha (2011)* | Korea | 10,050 | 56.35% | Missing | Missing | 5 years (range) | Metformin | Non-use of metformin | 1.RR=1.45 (1.21,1.74) | 6 |
| Imfeld (2012) | UK | 14,172 | 68.98% | 80.7 (6.7) | 80.7 (6.7) | 10 years (range) | Metformin, SU, TZD, or insulin | Non-use of corresponding drug | 1. Metformin: RR=1.25 (1, 1.56) | 7 |
|  |  |  |  |  |  |  |  |  | 2. SU: RR=0.906 (0.76,1.07) |  |
|  |  |  |  |  |  |  |  |  | 3. Insulin: RR=f0.73 (0.56,0.99) |  |
|  |  |  |  |  |  |  |  |  | 4. TZD: RR=0.93 (0.59,1.43) |  |
| Sluggett (2019) | Finland | 29,412 | 59.82% | 80.12 (6.15) | 80.12 (6.15) | 6 years (range) | Metformin | Non-use of metformin | 1. RR=0.99 (0.94,1.05) | 8 |

AD: Alzheimer's disease, CI: confidence intervals, MCI: mild cognitive impairment, RR: relative risks, SD: standard deviation, 2, SU: sulfonylureas, TZD: Thiazolidinediones

*nested case-control study

**Supplemental Text**

To convert odds ratios to relative risks, we used the following formula, and we set the non-exposed prevalence to 6.5% (22-24):

$RR=\frac{OR}{\left( 1-r \right)+\left( r*OR \right)}$

RR=relative risk; OR=odds ratio; r=the prevalence of the outcome in the reference group.

However, we used HR and RR for this study as the prevalence of the outcome is low (<10%), meaning that the value of RR and HR will be very similar.

Database:
Embase <1974 to 2023 November>

| **#** | **Query** | **Results from 21 Nov 2023** |
| --- | --- | --- |
| 1 | alzheimer disease/ or dementia/ | 360,443 |
| 2 | dement*.tw. | 209,127 |
| 3 | mild cognitive impairment/ | 37,765 |
| 4 | mild cognitive impair*.tw. | 37,197 |
| 5 | alzheimer*.tw. | 251,460 |
| 6 | cognitive decline.tw. | 49,465 |
| 7 | cognitive deterioration.tw. | 3,623 |
| 8 | 1 or 2 or 3 or 4 or 5 or 6 or 7 | 481,347 |
| 9 | diabetes mellitus/ | 712,998 |
| 10 | diabet*.tw. | 1,189,557 |
| 11 | 9 or 10 | 1,364,029 |
| 12 | 8 and 11 | 30,728 |
| 13 | Clinical trial/ | 1,075,058 |
| 14 | Randomized controlled trial/ | 792,468 |
| 15 | Randomization/ | 98,963 |
| 16 | Placebo/ | 404,985 |
| 17 | Randomi?ed controlled trial$.tw. | 330,504 |
| 18 | Rct.tw. | 54,977 |
| 19 | Random allocation.tw. | 2,618 |
| 20 | Randomly allocated.tw. | 46,129 |
| 21 | Allocated randomly.tw. | 2,984 |
| 22 | (allocated adj2 random).tw. | 955 |
| 23 | Placebo$.tw. | 370,443 |
| 24 | Prospective study/ | 893,698 |
| 25 | 13 or 14 or 15 or 16 or 17 or 18 or 19 or 20 or 21 or 22 or 23 | 2,021,436 |
| 26 | Case study/ | 97,839 |
| 27 | Case report.tw. | 548,983 |
| 28 | Abstract report/ or letter/ | 1,306,704 |
| 29 | or/26-28 | 1,938,104 |
| 30 | 25 not 29 | 1,971,892 |
| 31 | 12 and 30 | 2,113 |
| 32 | case control study/ | 209,304 |
| 33 | retrospective study/ | 1,517,884 |
| 34 | cohort analysis/ | 1,072,932 |
| 35 | prospective study/ | 893,698 |
| 36 | longitudinal study/ | 200,151 |
| 37 | case control stud*.tw. | 170,775 |
| 38 | retrospective stud*.tw. | 362,206 |
| 39 | cohort stud*.tw. | 477,961 |
| 40 | prospective stud*.tw. | 323,795 |
| 41 | longitudinal stud*.tw. | 128,536 |
| 42 | 32 or 33 or 34 or 35 or 36 or 37 or 38 or 39 or 40 or 41 | 3,439,332 |
| 43 | 12 and 42 | 6,851 |
| 44 | 31 or 43 | 8,620 |
| 45 | (exp animal/ or animal.hw. or nonhuman/) not (exp human/ or human cell/ or (human or humans).ti.) | 7,216,434 |
| 46 | 44 not 45 | 8,554 |
| 47 | risk/ | 509,057 |
| 48 | risk.tw. | 4,046,483 |
| 49 | incidence/ | 581,366 |
| 50 | incidence.tw. | 1,344,849 |
| 51 | incident/ | 5 |
| 52 | incident.tw. | 142,062 |
| 53 | 47 or 48 or 49 or 50 or 51 or 52 | 5,141,492 |
| 54 | 46 and 53 | 5,612 |
| 55 | limit 54 to dd=20230424-20231117 | 124 |

Medline

Database:
Ovid MEDLINE(R) ALL <1946 to November 2023>

| **#** | **Query** | **Results from 21 Nov 2023** |
| --- | --- | --- |
| 1 | Dementia/ | 62,706 |
| 2 | dement*.tw. | 145,885 |
| 3 | mild cognitive impairment.tw. | 24,168 |
| 4 | Alzheimer Disease/ | 121,414 |
| 5 | alzheimer*.tw. | 187,412 |
| 6 | cognitive decline.tw. | 33,092 |
| 7 | cognitive deterioration.tw. | 2,300 |
| 8 | 1 or 2 or 3 or 4 or 5 or 6 or 7 | 318,911 |
| 9 | diabetes mellitus/ or diabetes mellitus, type 1/ or diabetes mellitus, type 2/ | 379,211 |
| 10 | diabet*.tw. | 784,255 |
| 11 | 9 or 10 | 827,793 |
| 12 | 8 and 11 | 13,930 |
| 13 | Randomized controlled trials as Topic/ | 165,379 |
| 14 | Randomized controlled trial/ | 603,829 |
| 15 | Random allocation/ | 107,042 |
| 16 | Clinical trial/ | 539,085 |
| 17 | exp Clinical Trials as Topic/ | 386,164 |
| 18 | 13 or 14 or 15 or 16 or 17 | 1,315,080 |
| 19 | (clinic$ adj trial$1).tw. | 492,834 |
| 20 | Placebos/ | 35,934 |
| 21 | Placebo$.tw. | 251,278 |
| 22 | Randomly allocated.tw. | 37,367 |
| 23 | (allocated adj2 random).tw. | 841 |
| 24 | 19 or 20 or 21 or 22 or 23 | 737,117 |
| 25 | 18 or 24 | 1,680,508 |
| 26 | Case report.tw. | 409,780 |
| 27 | Letter/ | 1,236,413 |
| 28 | Historical article/ | 369,447 |
| 29 | Review of reported cases.pt. | 0 |
| 30 | Review, multicase.pt. | 0 |
| 31 | or/26-30 | 1,996,131 |
| 32 | 25 not 31 | 1,642,223 |
| 33 | Case-Control Studies/ | 330,738 |
| 34 | case control.tw. | 157,681 |
| 35 | Retrospective Studies/ | 1,160,048 |
| 36 | retrospective stud*.tw. | 229,802 |
| 37 | Cohort Studies/ | 334,841 |
| 38 | cohort stud*.tw. | 332,052 |
| 39 | Prospective Studies/ | 673,101 |
| 40 | prospective stud*.tw. | 211,163 |
| 41 | Longitudinal Studies/ | 168,101 |
| 42 | longitudinal stud*.tw. | 99,947 |
| 43 | 32 or 33 or 34 or 35 or 36 or 37 or 38 or 39 or 40 or 41 or 42 | 4,058,009 |
| 44 | 12 and 43 | 3,531 |
| 45 | (exp animal/ or animal.hw. or nonhuman/) not (exp human/ or human cell/ or (human or humans).ti.) | 5,161,369 |
| 46 | 44 not 45 | 3,462 |
| 47 | Risk/ | 128,572 |
| 48 | risk.tw. | 2,793,541 |
| 49 | incidence/ | 303,293 |
| 50 | incidence.tw. | 929,261 |
| 51 | 47 or 48 or 49 or 50 | 3,528,079 |
| 52 | 46 and 51 | 2,361 |
| 53 | Dementia/ | 62,706 |
| 54 | dement*.tw. | 145,885 |
| 55 | mild cognitive impairment.tw. | 24,168 |
| 56 | Alzheimer Disease/ | 121,414 |
| 57 | alzheimer*.tw. | 187,412 |
| 58 | cognitive decline.tw. | 33,092 |
| 59 | cognitive deterioration.tw. | 2,300 |
| 60 | 53 or 54 or 55 or 56 or 57 or 58 or 59 | 318,911 |
| 61 | diabetes mellitus/ or diabetes mellitus, type 1/ or diabetes mellitus, type 2/ | 379,211 |
| 62 | diabet*.tw. | 784,255 |
| 63 | 61 or 62 | 827,793 |
| 64 | 60 and 63 | 13,930 |
| 65 | Randomized controlled trials as Topic/ | 165,379 |
| 66 | Randomized controlled trial/ | 603,829 |
| 67 | Random allocation/ | 107,042 |
| 68 | Clinical trial/ | 539,085 |
| 69 | exp Clinical Trials as Topic/ | 386,164 |
| 70 | 65 or 66 or 67 or 68 or 69 | 1,315,080 |
| 71 | (clinic$ adj trial$1).tw. | 492,834 |
| 72 | Placebos/ | 35,934 |
| 73 | Placebo$.tw. | 251,278 |
| 74 | Randomly allocated.tw. | 37,367 |
| 75 | (allocated adj2 random).tw. | 841 |
| 76 | 71 or 72 or 73 or 74 or 75 | 737,117 |
| 77 | 70 or 76 | 1,680,508 |
| 78 | Case report.tw. | 409,780 |
| 79 | Letter/ | 1,236,413 |
| 80 | Historical article/ | 369,447 |
| 81 | Review of reported cases.pt. | 0 |
| 82 | Review, multicase.pt. | 0 |
| 83 | or/78-82 | 1,996,131 |
| 84 | 77 not 83 | 1,642,223 |
| 85 | Case-Control Studies/ | 330,738 |
| 86 | case control.tw. | 157,681 |
| 87 | Retrospective Studies/ | 1,160,048 |
| 88 | retrospective stud*.tw. | 229,802 |
| 89 | Cohort Studies/ | 334,841 |
| 90 | cohort stud*.tw. | 332,052 |
| 91 | Prospective Studies/ | 673,101 |
| 92 | prospective stud*.tw. | 211,163 |
| 93 | Longitudinal Studies/ | 168,101 |
| 94 | longitudinal stud*.tw. | 99,947 |
| 95 | 84 or 85 or 86 or 87 or 88 or 89 or 90 or 91 or 92 or 93 or 94 | 4,058,009 |
| 96 | 64 and 95 | 3,531 |
| 97 | exp animals/ not humans.sh. | 5,173,282 |
| 98 | 96 not 97 | 3,462 |
| 99 | Risk/ | 128,572 |
| 100 | risk.tw. | 2,793,541 |
| 101 | incidence/ | 303,293 |
| 102 | incidence.tw. or incident/ or incident.tw. | 1,010,987 |
| 103 | 99 or 100 or 101 or 102 | 3,574,523 |
| 104 | 98 and 103 | 2,386 |
| 105 | limit 104 to up=20230424-20231120 | 253 |

CENTRAL

Total: 680

Search Name: DM Sys Review CENTRAL 20230424_1

Comment:

ID Search

#1 (dement*):ti,ab,kw (Word variations have been searched)

#2 MeSH descriptor: [Dementia] this term only

#3 (Alzheimer*):ti,ab,kw (Word variations have been searched)

#4 MeSH descriptor: [Alzheimer Disease] this term only

#5 ("mild cognitive impairment"):ti,ab,kw (Word variations have been searched)

#6 ("cognitive decline"):ti,ab,kw (Word variations have been searched)

#7 ("cognitive deterioration"):ti,ab,kw (Word variations have been searched)

#8 #1 or #2 or #3 or #4 or #5 or #6 or #7

#9 (diabet*):ti,ab,kw (Word variations have been searched)

#10 MeSH descriptor: [Diabetes Mellitus] this term only

#11 #9 or #10

#12 #8 and #11

#13 (risk):ti,ab,kw (Word variations have been searched)

#14 MeSH descriptor: [Risk] this term only

#15 (incidence):ti,ab,kw (Word variations have been searched)

#16 (incident):ti,ab,kw (Word variations have been searched)

#17 #13 or #14 or #15 or #16

#18 #12 and #17 in Trials

## Supplemental Table 4. PRISMA checklist

| **Section and Topic** | **Item #** | **Checklist item** | **Location where item is reported** |
| --- | --- | --- | --- |
| **TITLE** | | |  |
| Title | 1 | Identify the report as a systematic review. | 1 |
| **ABSTRACT** | | |  |
| Abstract | 2 | See the PRISMA 2020 for Abstracts checklist. | 2 |
| **INTRODUCTION** | | |  |
| Rationale | 3 | Describe the rationale for the review in the context of existing knowledge. | 3 |
| Objectives | 4 | Provide an explicit statement of the objective(s) or question(s) the review addresses. | 4 |
| **METHODS** | | |  |
| Eligibility criteria | 5 | Specify the inclusion and exclusion criteria for the review and how studies were grouped for the syntheses. | 5-6 |
| Information sources | 6 | Specify all databases, registers, websites, organisations, reference lists and other sources searched or consulted to identify studies. Specify the date when each source was last searched or consulted. | 5-6 |
| Search strategy | 7 | Present the full search strategies for all databases, registers and websites, including any filters and limits used. | Supplemental |
| Selection process | 8 | Specify the methods used to decide whether a study met the inclusion criteria of the review, including how many reviewers screened each record and each report retrieved, whether they worked independently, and if applicable, details of automation tools used in the process. | 5-6 |
| Data collection process | 9 | Specify the methods used to collect data from reports, including how many reviewers collected data from each report, whether they worked independently, any processes for obtaining or confirming data from study investigators, and if applicable, details of automation tools used in the process. | 5 |
| Data items | 10a | List and define all outcomes for which data were sought. Specify whether all results that were compatible with each outcome domain in each study were sought (e.g. for all measures, time points, analyses), and if not, the methods used to decide which results to collect. | 5-6 |
|  | 10b | List and define all other variables for which data were sought (e.g. participant and intervention characteristics, funding sources). Describe any assumptions made about any missing or unclear information. | 5-6 |
| Study risk of bias assessment | 11 | Specify the methods used to assess risk of bias in the included studies, including details of the tool(s) used, how many reviewers assessed each study and whether they worked independently, and if applicable, details of automation tools used in the process. | 6-7 |
| Effect measures | 12 | Specify for each outcome the effect measure(s) (e.g. risk ratio, mean difference) used in the synthesis or presentation of results. | 5,7 |
| Synthesis methods | 13a | Describe the processes used to decide which studies were eligible for each synthesis (e.g. tabulating the study intervention characteristics and comparing against the planned groups for each synthesis (item #5)). | 5-7 |
|  | 13b | Describe any methods required to prepare the data for presentation or synthesis, such as handling of missing summary statistics, or data conversions. | 5-7 |
|  | 13c | Describe any methods used to tabulate or visually display results of individual studies and syntheses. | 5-7 |
|  | 13d | Describe any methods used to synthesize results and provide a rationale for the choice(s). If meta-analysis was performed, describe the model(s), method(s) to identify the presence and extent of statistical heterogeneity, and software package(s) used. | 7 |
|  | 13e | Describe any methods used to explore possible causes of heterogeneity among study results (e.g. subgroup analysis, meta-regression). | 7 |
|  | 13f | Describe any sensitivity analyses conducted to assess robustness of the synthesized results. | 7 |
| Reporting bias assessment | 14 | Describe any methods used to assess risk of bias due to missing results in a synthesis (arising from reporting biases). | 6 |
| Certainty assessment | 15 | Describe any methods used to assess certainty (or confidence) in the body of evidence for an outcome. | 7 |
| **RESULTS** | | |  |
| Study selection | 16a | Describe the results of the search and selection process, from the number of records identified in the search to the number of studies included in the review, ideally using a flow diagram. | 8 |
|  | 16b | Cite studies that might appear to meet the inclusion criteria, but which were excluded, and explain why they were excluded. | Supplemental |
| Study characteristics | 17 | Cite each included study and present its characteristics. | Supplemental |
| Risk of bias in studies | 18 | Present assessments of risk of bias for each included study. | Supplemental |
| Results of individual studies | 19 | For all outcomes, present, for each study: (a) summary statistics for each group (where appropriate) and (b) an effect estimate and its precision (e.g. confidence/credible interval), ideally using structured tables or plots. | Supplemental |
| Results of syntheses | 20a | For each synthesis, briefly summarise the characteristics and risk of bias among contributing studies. | 9-11 |
|  | 20b | Present results of all statistical syntheses conducted. If meta-analysis was done, present for each the summary estimate and its precision (e.g. confidence/credible interval) and measures of statistical heterogeneity. If comparing groups, describe the direction of the effect. | 9-11; Supplemental |
|  | 20c | Present results of all investigations of possible causes of heterogeneity among study results. | 9-11; figures 1-4; Supplemental |
|  | 20d | Present results of all sensitivity analyses conducted to assess the robustness of the synthesized results. | Supplemental |
| Reporting biases | 21 | Present assessments of risk of bias due to missing results (arising from reporting biases) for each synthesis assessed. | Supplemental |
| Certainty of evidence | 22 | Present assessments of certainty (or confidence) in the body of evidence for each outcome assessed. | Supplemental |
| **DISCUSSION** | | |  |
| Discussion | 23a | Provide a general interpretation of the results in the context of other evidence. | 12-14 |
|  | 23b | Discuss any limitations of the evidence included in the review. | 15 |
|  | 23c | Discuss any limitations of the review processes used. | 15 |
|  | 23d | Discuss implications of the results for practice, policy, and future research. | 16-17 |
| **OTHER INFORMATION** | | |  |
| Registration and protocol | 24a | Provide registration information for the review, including register name and registration number, or state that the review was not registered. | 4 |
|  | 24b | Indicate where the review protocol can be accessed, or state that a protocol was not prepared. | 4 |
|  | 24c | Describe and explain any amendments to information provided at registration or in the protocol. | 4 |
| Support | 25 | Describe sources of financial or non-financial support for the review, and the role of the funders or sponsors in the review. | 17 |
| Competing interests | 26 | Declare any competing interests of review authors. | 17 |
| Availability of data, code and other materials | 27 | Report which of the following are publicly available and where they can be found: template data collection forms; data extracted from included studies; data used for all analyses; analytic code; any other materials used in the review. | 17 |

*From:* Page MJ, McKenzie JE, Bossuyt PM, Boutron I, Hoffmann TC, Mulrow CD, et al. The PRISMA 2020 statement: an updated guideline for reporting systematic reviews. BMJ 2021;372:n71. doi: 10.1136/bmj
